# Ideomotor Action: Evidence for Automaticity in Learning, but Not Execution

**DOI:** 10.3389/fpsyg.2020.00185

**Published:** 2020-02-14

**Authors:** Dan Sun, Ruud Custers, Hans Marien, Henk Aarts

**Affiliations:** Department of Psychology, Utrecht University, Utrecht, Netherlands

**Keywords:** action control, automaticiy, goal-directed behavior, ideomotor, implicit learning

## Abstract

Human habits are widely assumed to result from stimulus-response (S-R) associations that are formed if one frequently and consistently does the same thing in the same situation. According to Ideomotor Theory, a distinct but similar process could lead to response-outcome (R-O) associations if responses frequently and consistently produce the same outcomes. This process is assumed to occur spontaneously, and because these associations can operate in a bidirectional manner, merely perceiving or thinking of an outcome should automatically activate the associated action. In the current paper we test this automaticity feature of ideomotor learning. In four experiments, participants completed the same learning phase in which they could acquire associations, and were either explicitly informed about the contingency between actions and outcomes, or not. Automatic action selection and initiation were investigated using a free-choice task in Experiment 1 and forced-choice tasks in Experiment 2, 3a, and 3b. An ideomotor effect was only obtained in the free-choice, but not convincingly in the forced-choice tasks. Together, this suggests that action-outcome relations can be learned spontaneously, but that there may be limits to the automaticity of the ideomotor effect.

## Introduction

Habits are often regarded to be the result of stimulus-response (S-R) associations that are assumed to be formed if people repeatedly and consistently perform the same behavior in the same situation, often because there is an incentive to do so (Wood and Rünger, 2016). As a consequence, the situation may trigger the associated response in an automatic fashion, leading to habitual behavior that is no longer guided by deliberative processes (Aarts and Dijksterhuis, 2000), but controlled by the environment. A relevant but distinct line of research proposes a similar mechanism in which behaviors can become associated with the situations or events that *follow* actions: Ideomotor theory proposes that if a behavioral response is repeatedly and consistently followed by the same perceptual outcome, thinking about or activating the mental representation of that outcome can to a certain extent prepare or trigger the behavior through bi-directional response-outcome (R-O) associations. This mechanism of ideomotor action has been used to explain various instances in which the environment triggers behaviors in an automatic fashion, such as mimicry, or behavior from affordances (Iacoboni, 2008; Custers and Aarts, 2010).

Ideomotor-action could be relevant to the understanding of habitual behavior in at least two ways. First, it may help to understand how the environment could trigger behaviors that look like habits, but may not be the result of classic habit formation processes (i.e., not resulting from S-R associations). Second, it may help to understand the implementation of seemingly abstract S-R associations. That is, many behaviors that are regarded as habits (reading the newspaper on Saturday morning, having coffee after dinner, reading a book before going to sleep) are not directly represented at the motor level, but representations include a rich collection of experiences of the consequences of executing the behavior and allow for an abstract representation of the behavior. Research indeed suggests that people represent behaviors in a hierarchical way, in which more abstract representations of the behavior are often the outcomes of the lower-level actions that produced them (Vallacher and Wegner, 1987; Kruglanski et al., 2002; Cooper and Shallice, 2006). Representing behaviors in terms of their outcomes may therefore help to produce the same behavioral outcome (e.g., reading the newspaper) under slightly different conditions (e.g., picking up the paper from a slightly different location on the doormat each time and finding an empty chair to read it; Powers, 1973; Custers and Aarts, 2010).

Although action-outcome representations may be indispensable for human behaviors, and especially goal-directed actions, it is less clear how these associations are acquired. Moreover, although contemporary approaches to ideomotor action (Hommel, 2013) assume that bi-directional R-O associations could trigger responses in an automatic fashion, there are few rigorous tests that demonstrate this. In the present paper we put the automaticity in the formation and execution of ideomotor action within the classic ideomotor paradigm to the test. We first review current evidence for the automatic nature of ideomotor action and evidence for spontaneous ideomotor learning. We then investigate whether or not learning relations between actions and outcomes can occur spontaneously, by merely executing actions and observing following events, and without specific instructions. Three different ideomotor tests are used to gain insight in the degree to which potentially resulting ideomotor actions are automatic.

### Ideomotor Theory

The notion of ideomotor action dates back to the 19th century (Carpenter, 1852; Lotze, 1852; James, 1890), aiming to explain how thought can trigger action (for reviews see, Stock and Stock, 2004; Shin et al., 2010). The central idea of early ideomotor theory was that merely envisioning an action triggers that action to a certain extent (James, 1890), even in the absence of a conscious intention to act (Ansfield and Wegner, 1996). Embracing the idea that thinking of an action includes envisioning its anticipated outcomes, Greenwald (1970) proposed that ideomotor action relies on bi-directional R-O associations. That is, thinking about an actions involves thinking about the perceptual experiences that have become associated with particular motor programs (see also., Zwaan and Taylor, 2006). While such associations enable response selection based on outcomes of actions (i.e., goal-directed behavior), the strong version of ideomotor theory (see Shin et al., 2010) holds that once the association is formed, thinking (ideation) of an outcome, or merely perceiving a related stimulus, is enough to trigger the associated action. This backward activation appears to be a robust and general phenomenon which has been observed for many different action and stimuli, such as auditory stimuli (e.g., Elsner and Hommel, 2001), faces (Herwig and Horstmann, 2011), locations (Hommel, 1993), and letters (Ziessler and Nattkemper, 2002; Hommel et al., 2003).

In the last two decades, the Theory of Event Coding (TEC) (Hommel et al., 2001) has revived interest in ideomotor action, by providing a cognitive-perceptual framework for understanding these effects. This framework holds that both actions and their perceived sensory effects are cognitively represented in a similar distributed fashion and that their feature codes become intricately linked in action-stimulus representations that contain information about both. As these representations can be used bi-directionally, observing or thinking of an outcome activates the representation of the corresponding action, explaining phenomena such as mimicry (Iacoboni, 2008) action priming (Dijksterhuis and Bargh, 2001), and goal priming (Custers and Aarts, 2010). According to TEC, representations of effects and basic motor movement already become intertwined in early infancy (Hommel et al., 2001; Heyes, 2010). It appears, then, that R-O associations emerge spontaneously as a result of acting and observing, giving rise to representations that can drive behavior in an automatic, habit-like fashion.

### Ideomotor Research

Following Greenwald (1970), tests of ideomotor learning typically contain two-phases: An acquisition phase in which action-outcome associations are acquired, and a test phase that tests whether these stimuli (i.e., outcomes) facilitate associated actions. In a classic study, Elsner and Hommel (2001) had participants freely choose in the first phase (i.e., free-choice acquisition phase) between left and right key presses that were each consistently followed by a specific tone (high or low pitch). Importantly, participants were explicitly informed that the tones were irrelevant to the task. In the second phase (forced-choice test phase, Experiments 1a, 1b), participants had to press left or right keys preceded by the tones that mapped on the earlier learned responses (non-reversal group), whereas for the other group the Response-Outcome mapping was reversed (reversal group). Results showed that actions were performed faster when the mapping was consistent with that in the acquisition phase, rather than reversed. Follow-up experiments (Experiments 2–4) revealed a similar consistency effect in a free-choice test phase that required subjects to press left and right keys randomly: Actions that were consistent with the Response-Outcome mapping were more frequently selected after the tones, showing a response bias in free choice as a result of outcome priming.

Later studies have systematically compared the effects of free- and forced-choice learning phases. Herwig et al. (2007) used a forced choice test-phase in which participants were allocated to a non-reversal or reversal group. They found that effects of ideomotor learning between actions and resulting outcomes only occurred when participants voluntarily selected actions in the learning phase (free-choice learning), and not when the required responses were forced by cues (forced-choice learning). These findings suggest that participants more readily represented the stimuli (tones) as outcomes of their actions when they engaged in free-choice learning, whereas merely responding to cues did not produce such a psychological process. Hence, even though actions were followed by stimuli in exactly the same way in free- and forced-choice learning phases, the stimulus information appears to have been encoded differently during learning.

Subsequent work by Pfister et al. (2011) suggested that it may not be the encoding in the acquisition phase, though, that makes the difference, but rather the mode in which people control their behaviors in the test phase. Using a free-choice test phase, they found evidence for ideomotor effects, regardless of whether learning took place in a free- or a forced choice phase. They concluded that ideomotor learning takes place whenever actions are followed by events, regardless of the acquisition task, but that participants need to be engaged in “intention-based control” in the test phase (that is, selecting outcome-related actions), for ideomotor effects to arise. This would suggest that while learning of habitual action-outcome relations may be spontaneous, it may be conditional on a certain mind set or task set (i.e., conditional automaticity; see Aarts and Dijksterhuis, 2000).

### Instruction Effects

Although the research discussed above suggests that ideomotor learning occurs spontaneously whenever events follow actions, this “spontaneous learning” always occurs within the experimental setting. As it happens, though, task instructions in the acquisition phase often explicitly mention the presence of outcomes in the task, stating that they are irrelevant and should be ignore (e.g., Elsner and Hommel, 2001). Whilst it is not always clear which exact instructions are provided in the acquisition phase in ideomotor research, Eder and Dignath (2017) have recently demonstrated in a task in which learning and testing of ideomotor action are intertwined, that such task instructions matter a lot. Based on recent insights in the power of instruction effects (see Liefooghe et al., 2018), Eder and Dignath provided instructions to ignore, attend, learn, or intentionally produce action outcomes in one combined learning/test phase. Results showed that instructions affect the task set with which action-stimulus relations are learned (Custers and Aarts, 2011), but that unlike the learning and intention instructions, instructions to ignore or attend to outcomes did not lead to ideomotor learning, at least not in this experimental setting.

In the present paper, we investigate whether ideomotor learning occurs spontaneously in the standard two-phase paradigm with auditory stimuli. In four studies, we manipulated instructions in a free-choice learning phase, either saying nothing at all about tones that followed actions, or emphasizing their relationship in terms of actions and outcomes. All experiments used a free-choice acquisition phase, as previous research suggests that action-outcome relations are more strongly acquired and subsequently used (Herwig et al., 2007; Pfister et al., 2011). Given the complexity of obtaining clear and reliable ideomotor effects, and in order to gain more insight in what is learned in the acquisition phase, we employed three different ideomotor tests in four separate experiments. In Experiment 1, we used a free-choice test phase, as earlier work has suggested that ideomotor effects are most likely to occur under such conditions (e.g., Pfister et al., 2011). However, as the free-choice ideomotor test is - by definition - open to influences of conscious deliberation and choice, we follow up in Experiment 2, 3a, and 3b with a forced-choice ideomotor test. While Experiment 2 used a 2-block design where participants received opposite instructions on the different blocks that forced them to react to outcome stimuli either in line with the acquired action-outcome mapping, or the opposite mapping, Experiment 3a and 3b used an interference paradigm with imperative cues (presented together with outcome stimuli) to force people’s choice on trial level. These forced-choice ideomotor tests would provide stronger evidence for the automatic initiation of actions than the free-choice test, with Experiment 3a and 3b being the least susceptible to alternative explanations. As such, the current line of experiments not only tests, but also aims to verify the automatic nature, of potential ideomotor actions arising from spontaneous ideomotor learning.

## Experiment 1: Free-Choice Ideomotor Test

### Method

#### Participants and Design

Sample sizes on previously published ideomotor learning studies which varied from 12 (e.g., Kühn et al., 2009, Experiment 1) to 20 participants per condition (e.g., Herwig and Waszak, 2009, Experiment 1–3), and given the fact that small sample sizes can counterintuitively inflate effect size, we decided prior to data collection to test at least 20 participants per condition in each experiment.

Fifty participants took part in the experiment in exchange for a small monetary payment or extra course credits. Participants with attention-related disorders or those who were on related medication were excluded beforehand. The experimental design consisted of one between-subjects factor: Instructions (No-Instructions vs. Instructions). After signing the informed consent, participants were randomly assigned to either the No-Instructions condition or the Instructions condition.

Data of one participant were lost because of a technical issue, and two participants were excluded due to the unbalanced proportion of key presses during the acquisition phase (outside of the range of a left-to-right ratio of 40 to 60%), which was defined before data collection. Data of the remaining 47 participants (No-Instructions condition: *n* = 23, Instructions condition: *n* = 24) were included in the analyses [37 females, mean age: 24 years (18–30 years), no left-handed and 2 ambidextrous participants].

#### Procedure

Participants were told that they would perform two tasks on a computer and were asked to read the instructions carefully. The present study used the same design as the third experiment of Elsner and Hommel (2001), consisting of an acquisition phase and a test phase. Both phases featured a Go – No-Go paradigm, and the auditory stimuli following responses in the acquisition phase [i.e., a low tone (400 Hz) and a high tone (800 Hz)] were presented again in the test phase upon which participants were to freely choose a left or a right response. After the acquisition phase, they continued with the second task (i.e., the test phase).

After the two main phases, participants filled out a short questionnaire that tested their knowledge about Response-Outcome mappings acquired in the learning phase and measured the representation levels on four hierarchically different levels of self-causation (i.e., association, prediction, causality, and agency level of Response-Outcome relations, see below) to check whether the instructions induced the desired processing goals differently. Response-Outcome mappings were counterbalanced among the participants. That is, for half of the participants, the left key was followed by the high tone and the right key by the low tone (Response-Outcome mapping A), whereas for the other half, the opposite key-tone mappings (Response-Outcome mapping B) were used.

#### Acquisition Phase

After general task instructions, all participants read the following specific instruction for the acquisition phase:

*“In this part you have to press a key with your left or right index finger, depending on the instructions on the screen: If you see “*<<>>*”, you can choose yourself to press the left key (“z”), or the right key (“/”). You can choose freely, but try, on average, to press left and right equally often. If you see “xxxx,” however, you should not press any key.”*

Participants in the Instructions condition were then given detailed additional information about the R-O mappings – which depended on the counterbalancing of the mapping – and were provided with processing goals through descriptions of the relationship between the responses and their outcomes in ascending levels of self-causation (i.e., from associative, predictive, to causal) in the acquisition phase:

“Pressing your left key is associated with a High [Low] tone and pressing your right key with a Low [High] tone. This means that upon pressing your left key you can predict a High [Low] tone and upon pressing your right key a Low [High] tone. In other words: pressing your left key causes a High [Low] tone and pressing your right key causes a Low [High] tone.”

It is important to keep in mind that in the No-Instructions condition the tones are just stimuli that consistently followed key-presses, without any related mention about the occurrence of the tones, and that in the Instructions condition the stage was set for processing the tones as outcomes of self-chosen actions.

**FIGURE 1:**
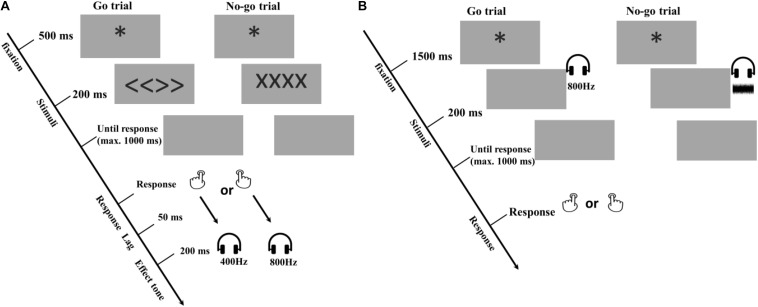
Procedure of Experiment 1. **(A)** acquisition phase for all the experiments. **(B)** free choice ideomotor test phase of Experiment 1. In the present example of the acquisition phase, the left response is always followed by a low tone (i.e., 400 Hz); whereas the right response is always followed by a high tone (i.e., 800 Hz), but these mappings were counterbalanced.

The trial procedures of the acquisition phase are depicted in Figure 1A. Each trial of the acquisition phase started with a fixation asterisk (^∗^) for 500 ms on the middle of the screen, followed by a 200-ms Go (i.e., “<<>>”) or No-Go (i.e., “xxxx”) signal. Participants were asked to press the left or right key freely as soon as they saw the Go signal and were asked not to respond in No-Go trials. The program waited up to 1,000 ms for a response. On Go trials, reaction times over 1,000 ms were treated as omissions and responses faster than 100 ms as anticipations. Only reaction times in the valid range (100–1000 ms) triggered the contingent tone, which started after a 50-ms lag from the onset of the keypress and was presented for 200 ms. Incorrect trials (i.e., omissions, anticipations, and responses to No-Go trials) were recorded, and were signaled to the participant by a 1000-ms warning messages on the screen saying: “too slow”, “too fast,” or “No-Go trial, respectively. All incorrect trials were repeated in random order by the end of the first task. Participants had to redo all the incorrect trials until all required responses were valid.

The acquisition phase consisted of three practice trials and 300 valid trials, divided into 10 blocks. Every two blocks, there was a 10 s break, during which participants were informed about how often they had pressed the left and right keys. In the Instructions condition, the extra processing information about the Response-Outcome mappings was also repeated (e.g., “Each specific key causes a specific tone. The left key causes a High tone and the right key causes a Low tone”).

#### Test Phase

The test phase was similar to the acquisition phase, also using the Go – No-Go paradigm. This time, however, two tones that previously served as outcomes were presented as cueing stimuli (see Figure 1B). Participants were instructed to press the left or right key randomly in response to the tone. In addition, as suggested by Elsner and Hommel (2001), to add response uncertainty and prevent participants from responding before the tone appeared, a novel sound (i.e., a 200-ms white noise signal) was presented in one third of the test trials, serving as a No-Go signal after which participants were to withhold their response. Each test trial started after an inter-trial interval of 1,500 ms with an asterisk on the center of the screen, followed by a 200-ms sound (i.e., a high tone, a low tone or a white noise signal), which were presented in a random order. Then the program waited up to 1,000 ms for an appropriate response. Response omissions and anticipations were defined in the same way as in the acquisition phase. However, this time no error message was presented and participants worked through six practice trials and 288 valid trials, divided into 8 blocks, including 96 No-Go trials in total. Again, every two blocks, there was a 10 s break. This time no extra information about the Response - Outcome mappings was provided during the break.

#### Manipulation Check of R-O Mappings

After the test phase, participants answered two questions that tested their knowledge about the relationship between the responses (i.e., left/right key presses) and the corresponding outcomes (i.e., low/high tones) in the acquisition phase, to check whether participants were able to report which tone followed which response. There were four answer options to each mapping question. For instance, when asked: “Which tone did the left key press produce?”, response option were: (1) the left key press produced the High tone, (2) the left key press produced the Low tone, (3) the left key press produced both tones, (4) the left key press was irrelevant to the tones” (see Supplementary Appendix S1 for more details).

#### Manipulation Check of Instructions

Subsequently, participants filled out a questionnaire designed to measure changes in the representation of the response-outcome relations as a result of the instructions manipulation. The questionnaire probed the four levels of the hierarchical representation used in the Instructions condition (i.e., association, prediction, causality, and agency level of Response-Outcome relations). Specifically, for each level, three items probed representations using a 9-point scale. The complete questionnaire can be found in the Appendix (see Supplementary Material). A difference between instruction conditions on these measures would indicate that the manipulation changed the way in which participants represented the response-outcome relations.

#### Data Analysis Plan

Data were analyzed using R 3.5 (R Core Development Team, 2014). Visualizations of raw data points were built with the raincloud plots (Allen et al., 2018). ANOVA’s were calculated using the aov_ez function and Type III sums of squares (afex package Version 0.22–1 in R) (Singmann et al., 2016). When assumptions of sphericity were violated Greenhouse-Geisser (GG) correction was utilized in the ANOVA model. In this case, we reported uncorrected degrees of freedom and corrected *p*-values. To further draw conclusions about the support of null effects, we also calculated Bayesian factors (BFs) with the default prior setting in JASP (version 0.9, JASP Team 2018) (van Doorn et al., 2019). The advantage of BFs is that it quantifies evidence in favor of one (e.g., null) hypothesis compared to another (e.g., alternative) hypothesis given the observed data.

### Results

#### Acquisition Phase

First, we excluded all acquisition trials with anticipations (No-Instructions: 0.01%, Instructions: 0.01%) and omissions (No-Instructions: 0.04%, Instructions: 0.09%). Failures to withhold responses on the No-Go trials were calculated and all participants fell below the pre-set criteria of less than 20% (No-Instructions: 2.89%, Instructions: 2.55%). After that, response proportions (left/right keypress) were calculated. To make sure the participants had followed the general instruction to press the left and right key randomly but equally often, participants with proportions outside the 40% to 60% range were excluded (see section “Participants and Design”). The mean left/right response proportions were equal in each condition – No-Instructions condition: 49.9% vs. 50.1%; Instructions condition: 49.6% vs. 50.4%.

The mean RTs of the participants did not differ between the No-Instructions, *M* = 362.94 ms, *SD* = 60.24 ms, and Instructions condition, *M* = 362.41 ms, *SD* = 39.01, *F*(1,45) = 0.00, *p* = 0.97. The mean RTs of right responses, *M* = 360.75 ms, *SD* = 52.40 ms, were marginally faster than the mean RTs of left responses *M* = 364.59 ms, *SD* = 48.49 ms, *F*(1,45) = 2.87, *p* = 0.10. This difference was not qualified by an interaction with the between-subjects factor Instructions, *F*(1, 45) = 0.75, *p* = 0.39.

#### Test Phase

Test trials with response anticipations (No-Instructions: 0.05%, Instructions: 0.06%) and omissions (No-Instructions: 1.31%, Instructions: 0.91%) were excluded from data analysis and the percentage of responses that were consistent with the previously acquired Response-Outcome mapping was calculated for each participant.

**FIGURE 2:**
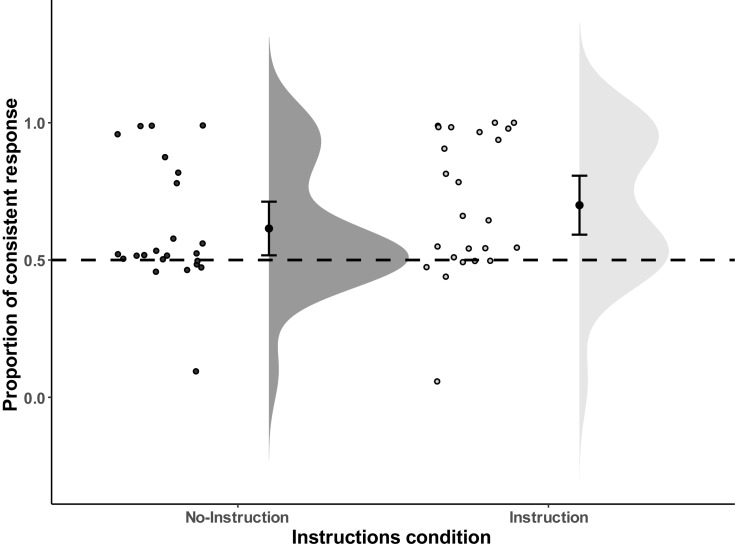
Distribution of the proportion of consistent responses of all the individual data points in Experiment 1. Error bars represent the 95% confidence intervals.

As expected, in the No-Instructions condition the mean proportion of consistent responses was significantly larger than chance (i.e., 50%), *M* = 61.49%, *SD* = 22.61%, *t*(22) = 2.44, *p* = 0.012 (one-tailed), Cohen’s dz^1^ = 0.508, and the Bayesian one sample *T*-Test resulted in BF_+__0_ = 4.80, which means that the data are approximately 4.8 times more likely to occur under H_+_ (i.e., proportion in consistent condition is higher than chance level, that is, larger than 50%), than under H_0_ (i.e., proportion in consistent condition is at chance level). This result indicates moderate evidence in favor of H_+_. The same effect was observed for the Instructions condition: *M* = 69.98%, *SD* = 25.42%, *t*(23) = 3.85, *p* = 0.0004 (one-tailed), Cohen’s dz = 0.786, and the Bayesian one sample *T*-Test result is BF_+__0_ = 83.90, which indicates strong evidence in favor of H_+_. Finally, we tested whether instructions affected the proportion of consistent responses, but the direct comparison between the two conditions did not reveal any significant difference, *t*(45) = −1.21, *p* = 0.23 (two-tailed), and the Bayesian Independent samples *T*-Test result equals (BF_01_) 1.91, which only slightly favors the null hypothesis (H_0_: The Instructions condition has no effect on response preference) over the alternative hypothesis (H_1_: the Instructions condition biases response selection). In sum, while there was very strong support for an ideomotor effect in the Instructions condition and substantial evidence in the No-Instructions condition, evidence for no difference between Instructions conditions was only anecdotal (see Figure 2 for distribution).

Furthermore, we compared RTs for consistent and inconsistent trials in both Instructions conditions. There was no difference between consistent (*M* = 502.42 ms, *SD* = 95.89 ms) and inconsistent trials (*M* = 503.64 ms, *SD* = 99.22 ms) in the No-Instructions condition, *t*(22) = −0.14, *p* = 0.894; nor between consistent (*M* = 510.68 ms, *SD* = 70.11 ms) and inconsistent trials (*M* = 506.64 ms, *SD* = 69.98 ms) in the Instructions condition, *t*(21) = 0.77, *p* = 0.453. The corresponding BF also indicates moderate evidence for the null hypothesis (H_0_: The reaction times are not different between consistent and inconsistent trials) over the alternative hypothesis (H_1_: The reaction times are different between consistent and inconsistent trials) in No-Instruction condition (BF_01_ = 4.535) and Instruction condition (BF_01_ = 3.448), respectively.

#### Manipulation Check of R-O Mappings

Most participants (85% in total) were able to explicitly report the correct mapping of responses and subsequent stimuli they were exposed to in the acquisition phase. Six people missed the response-stimulus mapping in the No-Instructions condition (6 out of 23), and only one participant failed in the Instructions condition (1 out of 24).

#### Manipulation Check of Instructions

In order to assess whether there were differences in how people represented the relation between responses and outcomes in the acquisition phase, the average of each of the three questions measuring association, prediction, causality, and agency was calculated. The mean scale ratings were analyzed as a function of Instructions conditions and as a function of representation level (i.e., the hierarchical levels explained before). Only a main effect of representation level was found, *F*(3,135) = 7.97, *p*_[GG]_ < 0.001, $\eta {}_{\text{p}}^{2}$ = 0.15, which merely showed that collapsed over Instructions conditions, there were significant differences in ratings between the four level of representation constructs (Table 1 and Supplementary Table S1.1 presents more details of the responses to the scales).

**TABLE 1:** Means and Standard deviations of the four different representation levels collapsed over Instructions conditions for all three experiments.

Representation level	Exp1: free	Exp2: block-based	Exp3: trial-based
Association	7.45 ± 2.18	6.90 ± 2.63	7.55 ± 2.20
Prediction	6.67 ± 2.86	6.99 ± 2.73	7.32 ± 2.53
Causality	6.99 ± 2.78	7.03 ± 2.75	7.60 ± 2.23
Agency	6.26 ± 2.92	6.16 ± 3.10	6.83 ± 2.75

*The means of each scale is relatively high, indicating that in both the Instructions and No-instructions condition participants processed the learning task in line with the Instructions manipulation.*

### Discussion

These results provide support for an ideomotor effect, in the sense that tones followed responses in the acquisition phase were more likely to evoke these responses in the test phase. Moreover, this effect occurred regardless of instructions about the relation between responses and tones, which demonstrates that ideomotor learning – at least in the current paradigm – unfolded spontaneously.

Although the ideomotor effect was observed within both instruction conditions, it appeared more pronounced in the instructions condition. Bayesian tests, however, revealed slightly more support for the absence of difference between the two conditions. While it cannot be ruled out that instructions can strengthen ideomotor learning, it is clear that instructions were not necessary for learning to occur in the acquisition phase. This finding is further corroborated by an absence of a difference in the representation-level checks.

While the observed ideomotor effect obtained in the test phase seems comparable in size with other ideomotor studies (c.f., Elsner and Hommel, 2001), the free choice test phase does not provide strong evidence for the automatic nature of the effect (i.e., that the responses are triggered automatically by the stimuli that served as outcomes in the acquisition phase), as this task allows for deliberate responses in the test phase as well. On closer inspection, the response data show a bimodal distribution, with the majority of people responding at chance level and a considerable amount of people demonstrating a very large bias, with some participants showing near perfect consistence with the mapping acquired in the acquisition phase. This could suggest that the observed effect was not so much produced by the tones triggering the corresponding actions in the test phase, but by some people deliberately responding in line with the mapping learned in the acquisition phase. We return to this issue in the general discussion.

To rule out these more deliberate sources of the compatibility effect and to investigate whether spontaneously learned action-outcome associations can cause outcome stimuli to trigger ideomotor action directly, Experiments 2, 3a, and 3b used a forced-choice task, in which responses required by imperative cues or instructions were accompanied by tones that – according to the mapping learned in the acquisition phase – should trigger either compatible or incompatible responses. While compatible and incompatible trials were presented in separate blocks in Experiment 2, they were intermixed in Experiments 3a and 3b.

## Experiment 2: Block-Based Interference Ideomotor Test

In Experiment 2, we used a block-based interference ideomotor test in which participants completed two test blocks. In the compatible block, participants received instructions to respond to tones that were compatible with the earlier acquired mapping. In the incompatible block, the instructions were reversed. The order of the two test blocks was counterbalanced across participants. We expected to observe significantly reduced RTs and lower error rates in compatible blocks compared to incompatible blocks.

### Method

#### Participants and Design

Fifty participants took part in the experiment in exchange for a small monetary payment or extra course credits. Participants with attention related disorders or those who were on related medication were excluded beforehand. Participants were randomly assigned to a cell of the 2 (Instructions: No-Instructions vs. Instructions) ^∗^ 2 (Compatibility: Compatible vs. Incompatible) mixed factorial design, with Compatibility as a within-participants variable. The order of the compatible and incompatible blocks was counterbalanced across participants.

Three participants were excluded due to the unbalanced proportion of key pressing during the learning phase, that is, the balanced left-to-right key ratio (i.e., 40–60%). Data of the remaining 47 participants (No-Instructions condition: *n* = 24 vs. Instruction condition: *n* = 23) were analyzed in the test phase [23 Females, mean age: 22 years (18–31 years), no left-handed and two ambidextrous participants].

#### Procedure

The procedure was similar to Experiment 1. After finishing the unchanged acquisition phase, participants came to the interference ideomotor task with the compatibility manipulated on the block level. With regard to the acquired R-O mapping, the response rule participants received on one block was compatible, whereas on the other block it was incompatible. For example, if the participant got the R-O mapping A (left key – high tone, right key – low tone), the compatible block meant that participants were asked to press left key when hearing a high tone, and right key for a low tone; while the response rule in the incompatible block was reversed, that is, pressing left key for a low tone, and right key for a high tone.

#### Acquisition Phase

The acquisition phase was as identical as the first task of Experiment 1 (see Figure 1A).

#### Test Phase

**FIGURE 3:**
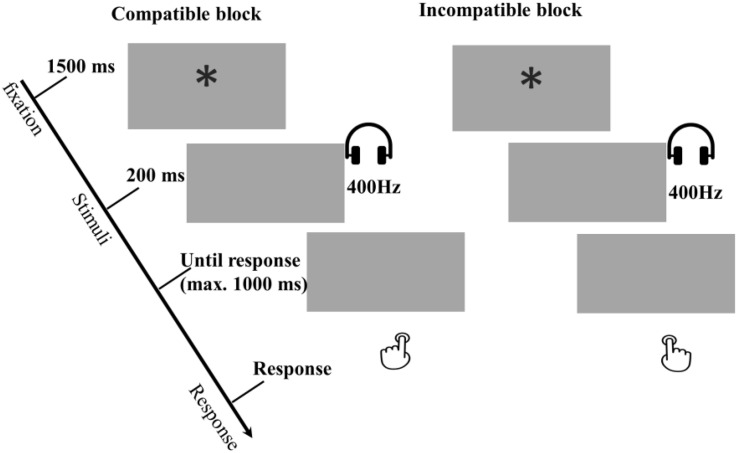
Examples of compatible and incompatible conditions in the Block-based interference test phase of Experiment 2. In these examples a low tone of 400 Hz was mapped to a left response. All other combinations were possible, but are not presented in this figure. During the task, participants were asked to respond to tones directly based on the response rule, and the orders between compatible and incompatible are counterbalanced between participants. The compatible and incompatible blocks are defined depending on the Response-Outcome mapping in the acquisition phase.

Both the compatible and the incompatible block, consisted of 4 sub-blocks of 24 trials (see Figure 3). The order of the blocks was counterbalanced between participants. Each trial began with a 1500-ms fixation with an asterisk (“^∗^”) centered in the screen, and then one of the two effect tones (i.e., the one learned in acquisition phase) was presented for 200 ms. The program would wait up to 1,000 ms to accept a response. On the first block, participants were instructed to respond according to either the compatible or incompatible response rule. Before switching to the second block with the opposite rule of responding, participants had to perform two example trials in which the responding requirements where explained as well as four practice trials without any clues.

#### Manipulation Check of R-O Mappings

The questions were the same as in Experiment 1.

#### Manipulation Check of Instructions

The questionnaire was the same as in Experiment 1.

### Results and Discussion

#### Acquisition Phase

Trials with response omissions (No-Instructions condition: 0.05%, Instruction condition: 0.11%) or anticipations (No-Instructions condition: 0.05%, Instructions condition: 0.05%) were excluded. After that, response proportions (left vs. right keypress) were calculated for each group. The mean left/right response proportions were equal in each condition (No-Instructions condition, 50.2% vs. 49.8%; Instruction condition: 49.6% vs. 50.4%).

The mean RTs of the participants did not differ between the No-Instructions, *M* = 374.38 ms, *SD* = 33.98 ms, and Instructions condition, *M* = 376.57 ms, *SD* = 37.73 ms, *F*(1,45) = 0.04, *p* = 0.83. The mean RTs of right responses *M* = 375.82 ms, *SD* = 33.81 ms, were not faster than the mean RTs of left responses *M* = 375.09 ms, *SD* = 37.83 ms, *F*(1,45) = 0.13, *p* = 0.72. There was also no interaction with the between-subjects factor Instructions, *F*(1,45) = 0.92, *p* = 0.34.

#### Test Phase

Participants who failed to meet the response criteria in the acquisition phase were excluded (3 participants), Furthermore, this time there were no trials with response anticipations (No-Instructions condition: 0%, Instructions condition: 0%), and trials with omissions (No-Instructions condition: 1.60%, Instructions condition: 1.22%) were excluded from data analysis.

##### Error rates

A 3-way mixed 2 (Instructions: No-Instructions vs. Instructions) ^∗^ 2 (Order: Compatible First vs. Incompatible First) ^∗^ 2 (Compatibility: Compatible vs. Incompatible) ANOVA yielded a main effect of Order, *F*(1,43) = 5.51, *p* = 0.02, $\eta {}_{\text{p}}^{2}$ = 0.11. Neither the main effect of Instructions, *F*(1,43) = 0.04, *p* = 0.85, nor that of Compatibility, *F*(1,43) = 0.00, *p* = 0.97, was significant. No significant interaction effects between Instructions ^∗^ Order *F*(1,43) = 0.99, *p* = 0.32, between Instructions ^∗^ Compatibility, *F*(1,43) = 0.25, *p* = 0.62, or between Instructions ^∗^ Order ^∗^ Compatibility, *F*(1,43) = 0.02, *p* = 0.89, were found. Only a 2-way interaction between Order and Compatibility, *F*(1,43) = 4.36, *p* = 0.04, $\eta {}_{\text{p}}^{2}$ = 0.09, was found showing that the direction of the Compatibility effect was different for the two Order conditions. However, the Compatibility effect was not significant in the Compatible first condition, *t*(43) = 1.46, *p* = 0.15, nor was the Compatibility effect significant in the Incompatible first condition, *t*(43) = −1.49, *p* = 0.14.

To further evaluate the evidence for the absence of a compatibility effect, the compatibility effect on error rates was calculated for all participants regardless of Instructions. If anything, errors showed a reversed compatibility effect (*M*_CE_ = −0.07324, *SD*_CE_ = 0.042) and the independent *T*-Test results, *t*(46) = −0.012, *p* = 0.51 (one-tailed), BF_0__+_ = 6.373, indicated moderate evidence for the null hypothesis (i.e., there is no difference between compatible and incompatible condition, namely, CE = 0) against the one-sided alternative hypothesis (i.e., the incompatible condition has more error rates than the compatible condition, namely, CE > 0).

Previous research tested the compatibility effect in a between-subjects design with a non-reversal and reversal group (e.g., Elsner and Hommel, 2001, Experiment 1a, 1b). In such a design, there is only one test block and participants just receive a compatible or incompatible response rule. To perform a comparable analysis on our date we zoomed in on the first block only, with Compatibility as a between-subjects factor.

**FIGURE 4:**
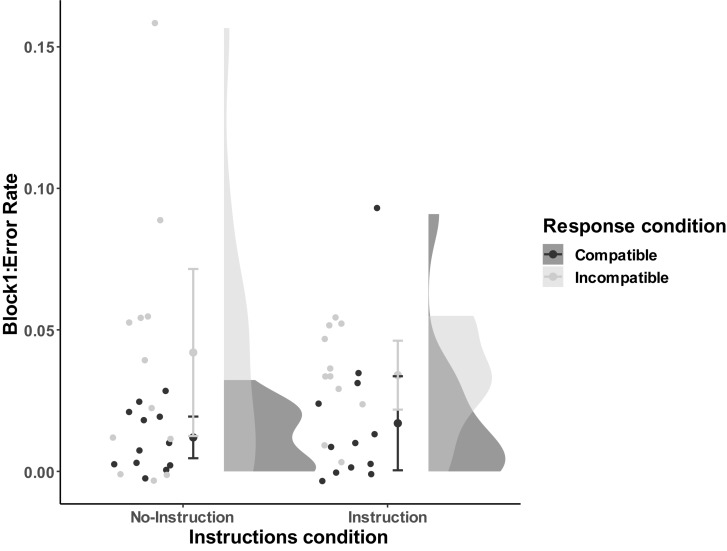
Distribution of error rates as a function of Compatibility and Instructions of block 1 in Experiment 2. The compatible and incompatible trials are defined depending on the Response-Outcome mapping in the acquisition phase. Error bar represent the 95% confidence interval.

For the first block, we conducted a 2-way between-subjects ANOVA (*M*_no–instruction_compatible_ = 0.012, *SD* = 0.012; *M*_no–instruction_incompatible_ = 0.042, *SD* = 0.046; *M*_instruction_compatible_ = 0.017, *SD* = 0.026; *M*_instruction_incompatible_ = 0.034, *SD* = 0.018). The results found a significant effect of compatibility, *F*(1,43) = 7.47, *p* = 0.009, $\eta {}_{\text{p}}^{2}$ = 0.15, but no main effect of Instructions, *F*(1,43) = 0.02, *p* = 0.90, nor an interaction, *F*(1,43) = 0.66, *p* = 0.42 (see Figure 4 for error rates in the first block visualized distribution).

##### Reaction times

**FIGURE 5:**
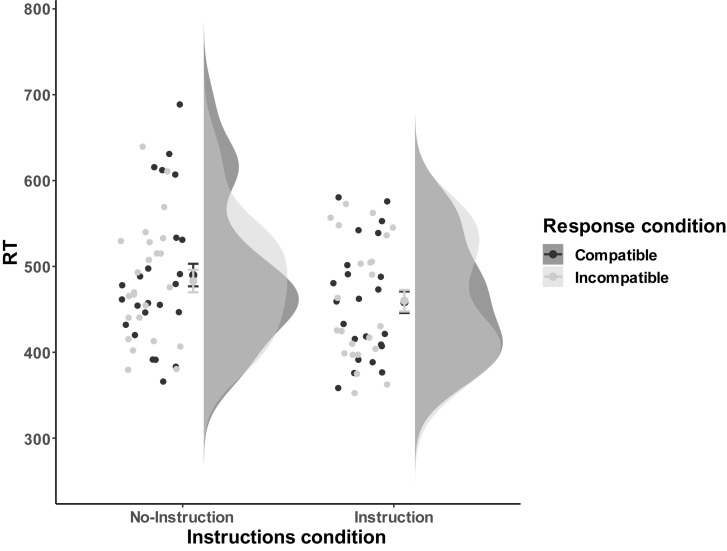
The response performance distribution of all the individual data points for each condition in Experiment 2. The compatible and incompatible trials are defined depending on the Response-Outcome mapping in the acquisition phase. Error bars represent the 95% confidence intervals.

Mean RTs for correct trials were subjected to a 3-way 2 (Instructions: No-Instructions vs. Instructions) ^∗^ 2 (Order: Compatible First vs. Incompatible First) ^∗^ 2 (Compatibility: Compatible vs. Incompatible) mixed measure ANOVA, that along with the between-participants factor Instructions and the within-participants factor Compatibility also included the counterbalancing between-participants factor Order. No main effects of Instructions, *F*(1,43) = 1.67, *p* = 0.20, Order, *F*(1,43) = 0.03, *p* = 0.88, and Compatibility, *F*(1,43) = 0.18, *p* = 0.67, were found. Furthermore, the Instruction ^∗^ Order, *F*(1,43) = 0.10, *p* = 0.75, Instruction ^∗^ Compatibility, *F*(1,43) = 0.46, *p* = 0.50, and Order ^∗^ Compatibility, *F*(1,43) = 0.65, *p* = 0.42, interactions were not significant, neither was the 3-way interaction, *F*(1,43) = 0.00, *p* = 0.99 (see Figure 5 for visualized distribution).

To further evaluate the evidence for the absence of a compatibility effect, the compatibility effect was calculated for all participants regardless of Instructions and Order. If anything, the compatibility effect was reversed, *M*_CE_ = −2.65 ms, *M*_CE_ = 42.34 ms, and the independent *T*-Test results, *t*(46) = −0.43, *p* = 0.665, BF_0__+_ = 8.54, provided relevant moderate evidence for the null hypothesis (i.e., there is no difference between compatible and incompatible condition, namely, CE = 0) against the one-sided alternative hypothesis (i.e., the reaction time in the incompatible condition is longer than the compatible condition, namely, CE > 0).

To further explore the data we zoomed in on the first block only, with Compatibility as a between-subjects factor, comparable to earlier ideomotor research. The RTs were subjected to a 2-way between-subjects ANOVA (*M*_no–instruction_compatible_ = 492.46 ms, *SD* = 81.76 ms; *M*_no–instruction_incompatible_ = 475.46 ms, *SD* = 73.38 ms; *M*_instruction_compatible_ = 454.18 ms, *SD* = 57.94 ms; *M*_instruction_incompatible_ = 459.18 ms, *SD* = 72.06 ms). Again, no significant results were found, Instructions, *F*(1,43) = 1.69, *p* = 0.20; Compatibility: *F*(1,43) = 0.08, *p* = 0.78; Interaction: *F*(1,43) = 0.28, *p* = 0.60.

#### Manipulation Check of R-O Mappings

Not all participants (only 60% correct, 28 out of 47) were able to explicitly report the correct mapping of actions and outcomes they were exposed to in the acquisition phase. In the No-Instructions condition, 10 out of 24 participants failed, either forming a reversed R-O mapping, or randomly guessing the R-O mapping. The Instructions condition has similar pattern, 9 out of 23 participants missed the correct R-O mapping rule. This number may be lower than in Experiment 1, though, as the test phase also featured the opposite mapping, which may have confused participants.

#### Manipulation Check of Instructions

In order to assess whether there were differences in how people represented the relation between responses and outcomes in the acquisition phase, the average of each three questions measuring association, prediction, causality, and agency was calculated. The 2 (Instructions condition: No-Instruction vs. Instructions) ^∗^ 4 Representation level ANOVA only found a main effect of Representation level, *F*(3,135) = 4.25, *p*_[GG]_ = 0.01, $\eta {}_{\text{p}}^{2}$ = 0.09, which merely showed that collapsed over Instructions conditions, there were significant differences in ratings between the four level of representation constructs (Table 1 presents more details of the responses to the scales).

#### Discussion

The block-based compatibility paradigm only provided limited support for an ideomotor effect. While no effects on RTs were found, participants made more errors on incompatible than compatible trials, though only on the first block. With no difference between instructions, this effect on errors at first glance seems to replicate the finding of Experiment 1, that ideomotor learning occurs spontaneously, also in the absence of instructions.

This compatibility effect – especially in the first block – could, however, also emerge as a result of a task switch (Monsell, 2003) that required participants who started with the incompatible block to use a new mapping, whereas participants in the compatible condition could still rely on the mapping that was learned in the acquisition phase. This effect should be less pronounced – or non-existing – in the second block, as participants in both order conditions would have to switch mappings. Note that an ideomotor effect based on an R-O association forged in the acquisition phase would predict a compatibility effect on the second block as well, as participants who entered the compatible after the incompatible block would benefit from the automatic responses triggered by the primes.

Evidence for a within-participants compatibility effect, however, was not obtained. A closer inspection of the pattern revealed that while participants who moved from a compatible to an incompatible block made more errors on the second block, showing a classic compatibility effect, participants who moved from the incompatible to the compatible block also made more errors on the second block. This suggests that the switch in instructions from block 1 to block 2 created more errors, regardless of whether the new rule was compatible or incompatible with the acquisition phase. This may indicate that people simply struggled to switch to a new response rule.

In order to rule out this possibility Experiment 3a and 3b were conducted, in which the compatibility effect was tested at trial level. This time, participants were instructed to react to imperative cues, but were at the same time presented with stimuli that had followed responses in the acquisition phase. These stimuli should interfere with participants’ responses if they are associated responses that are incompatible with the imperative cues. Such a trial-based interference ideomotor test would be the most rigorous test and cannot be regarded as a task-switch effect.

## Experiment 3: Trial-Based Interference Ideomotor Test

### Experiment 3a

#### Method

##### Participants and design

Sixty participants took part in the experiment in exchange for a small monetary payment or extra course credits. Participants with attention-related disorders or those who were on related medication were excluded beforehand. The experimental design consisted of one between-subjects factor: Instructions (No-Instructions vs. Instructions), and one within-subjects factor: Compatibility (Compatible vs. Incompatible). After signing the informed consent, participants were randomly assigned to either the Instructions condition or the No-Instructions condition.

Data of one participant were lost because of a technical issue, and five participants were excluded due to the unbalanced proportion of key presses during the learning phase (outside of the range of a left-to-right ratio of 40 to 60%), which was defined before data collection. Data of the remaining 54 participants (No-Instructions condition: n = 25 vs. Instructions condition = 29) were analyzed in the test phase [35 females, mean age: 23 years (18–37 years), 7 left-handed and 3 ambidextrous participants].

##### Stimuli and procedure

We used the same sounds as in Experiment 1, plus a standard Landolt “C” ring and its mirror image, as the target for the interference ideomotor task in the test phase. We selected these stimuli because they are clearly different form the arrow stimuli in the acquisition phase, making sure that imperative cues were not associated with responses (Muhle-Karbe and Krebs, 2012). Procedures were similar to Experiment 1, including an acquisition phase and a test phase.

##### Acquisition phase

The acquisition phase was as identical to the one used in Experiment 1 (see Figure 1A).

##### Test phase

**FIGURE 6:**
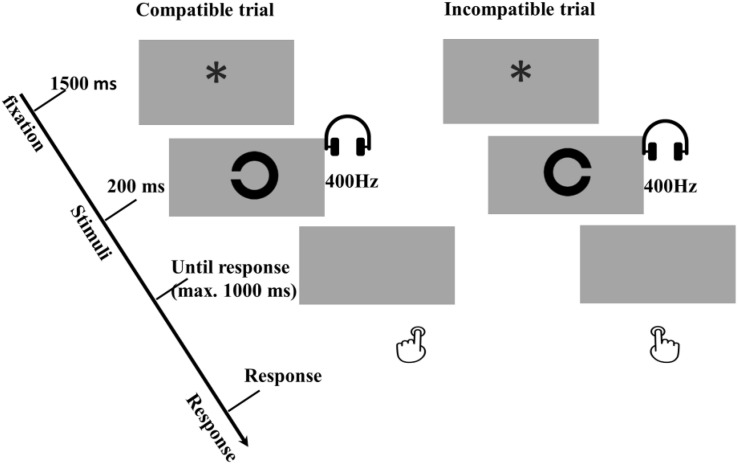
Examples of compatible and incompatible conditions in the trial-based interference test phase of Experiment 3a and 3b. In these examples a low tone of 400 Hz was mapped to a left response, which depending on the R-O mapping in the acquisition phase. All other combinations were possible, but are not presented in this figure. The main task is the orientation discrimination task with the tones as primes, and the compatible and incompatible are intermixed in trials level.

In the test phase participants were asked to perform an interference task, namely, the compatibility task, consisting of eight main blocks of 24 trials. Each trial started with a 1500-ms fixation (“^∗^”), and then one of the former effect sounds was simultaneously presented with the Landolt “C” (see Figure 6). The duration of the prime and the target were 200 ms and 250 ms, respectively. Participants were told to detect and respond to the opening direction of Landolt “C” ring as fast and accurately as possible. Pressing the left key (“z”) for a left opening, and the right key (“/”) for a right. The program waited up to 1,000 ms for a response. Response omissions and anticipations were defined in the same way as in the acquisition phase. There was no response feedback in the test phase.

Based on the R-O mapping in the acquisition phase, the test trials were categorized as a compatible trial when the to-be-executed response was the same as the response that was followed by the primed tone in the acquisition phase and incompatible trials when the to-be-executed response was the opposite of the response that was followed by the primed tone in the acquisition phase. For instance, if one had received the response – outcome mapping “left key – low tone, right key – high tone”, a trial was compatible when a left opening “C” ring was presented together with a low tone, and when a right opening “C” ring was presented with a high tone. A trial was incompatible when a left opening “C” ring accompanied by a high tone, and a right opening “C” ring with a low tone.

##### Manipulation check of R-O mappings

The questions were the same as in Experiment 1.

##### Manipulation check of instructions

The questionnaire was the same as in Experiment 1.

#### Data Analysis Plan

Analyses were similar to Experiment 1, RTs and error rates in the test phase were analyzed as a function of Instructions and Compatibility conditions.

#### Results

##### Acquisition phase

First, we excluded all acquisition trials with anticipations (No-Instructions: 0.09%, Instructions: 0.09%) and omissions (No-Instructions: 0.05%, Instructions: 0.08%). The remaining mean error rate for the No-Instructions condition was 4.78%, whereas for the Instructions condition it was 3.63%. After that, response proportions (left vs. right keypress) were calculated for each group. The mean left/right response proportions were equal in each condition (No-Instructions condition: 49.6% vs. 50.4%; Instructions condition: 49.8% vs. 50.2%).

The mean RTs of the participants did not differ between the No-Instructions, *M* = 344.61 ms, *SD* = 51.34 ms, and the Instructions condition, *M* = 358.07 ms, *SD* = 43.48 ms, *F*(1,52) = 1.10, *p* = 0.30. The mean RTs of right responses *M* = 349.00 ms, *SD* = 46.91 ms, were significantly faster than the mean RTs of left responses *M* = 354.67 ms, *SD* = 48.43 ms, *F*(1,52) = 7.03, *p* = 0.01, $\eta {}_{\text{p}}^{2}$ = 0.12. This effect was not qualified by an interaction with the between-subjects factor Instructions, *F*(1,52) = 0.60, *p* = 0.44.

##### Test phase

Participants who failed to meet the response criteria in the acquisition phase were excluded (five participants). Furthermore, trials with response anticipations (No-Instructions condition: 0.02%, Instructions condition: 0.036%) and omissions (No-Instructions condition: 0.0%, Instructions: 0.018%) were excluded from data analysis.

###### Error rates

**FIGURE 7:**
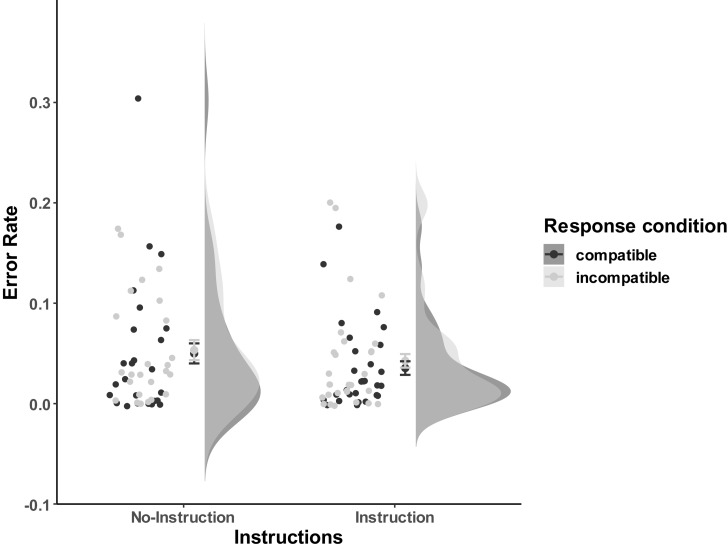
Distribution of error rates of Experiment 3a. The compatible and incompatible trials are defined depending on the Response-Outcome mapping in the acquisition phase. Error bars represent the 95% confidence intervals.

Error rates were analyzed based on all trials. As Figure 7 shows, participants were relatively accurate, and most of the error rates per condition were less than 10% (*M*_no–instruction_compatible_ = 0.050, *SD* = 0.070; *M*_no–instruction_incompatible_ = 0.053, *SD* = 0.054; *M*_instruction_compatible_ = 0.035, *SD* = 0.043; *M*_instruction_incompatible_ = 0.043, *SD* = 0.053). The 2 (Instructions: No-Instructions vs. Instructions) ^∗^ 2 (Compatibility: Compatible vs. Incompatible) mixed ANOVA did not reveal any significant effects [Instructions effect: *F*(1,52) = 0.74, *p* = 0.39; Compatibility: *F*(1,52) = 1.67, *p* = 0.20; Interaction: *F*(1,52) = 0.23, *p* = 0.63].

Thereafter, in further exploratory analyses, we calculated the compatibility effect on error rates by collapsing over the Instructions factor (*M*_CE_ = 0.0054, *SD*_CE_ = 0.029). An independent *T*-Test, *t*(53) = 1.34, *p* = 0.09, BF_0__+_ = 1.61, provided relevant moderate evidence for the null hypothesis (i.e., there is no difference between compatible and incompatible condition, namely, CE = 0) against the one-sided alternative hypothesis (i.e., the incompatible condition has more error rates than the compatible condition, namely, CE > 0).

###### Reaction times

**FIGURE 8:**
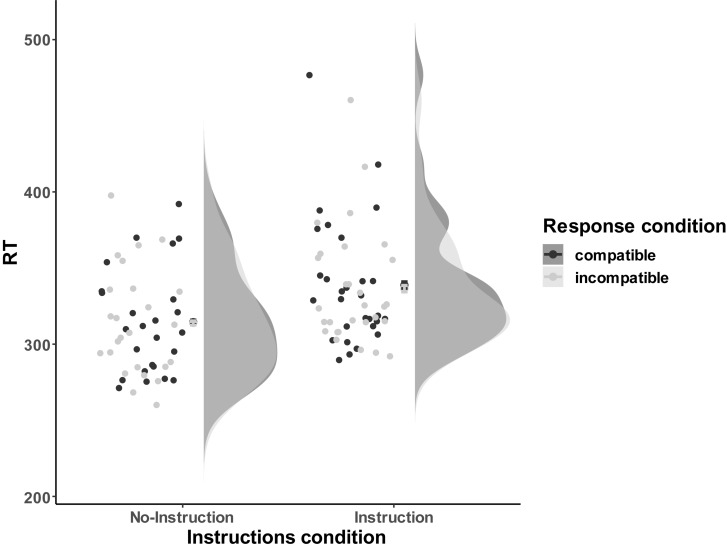
The response performance distribution of all the individual data points for each condition in Experiment 3a. The compatible and incompatible trials are defined depending on the Response-Outcome mapping in the acquisition phase. Error bars represent the 95% confidence intervals.

Reaction times (RTs) for remaining correct trials were aggregated over compatible and incompatible trials for each participant (see Figure 8, for visual distribution). Subsequently, the mean RTs and error rates were subjected to a 2 (Instructions: No-Instructions vs. Instructions) ^∗^ 2 (Compatibility: Compatible vs. Incompatible) ANOVA, with Instruction as between and Compatibility as within-subjects factor. RTs analysis did not reveal a significant compatibility effect, *F*(1,52) = 2.26, *p* = 0.14. Neither the effect of interaction reached significance *F*(1,52) = 0.91, *p* = 0.34, but we found a main effect of Instruction *F*(1,52) = 5.23, *p* = 0.03, $\eta {}_{\text{p}}^{2}$ = 0.09, indicating that participants in the Instructions condition were overall slower to respond (*M*_No–Instrucitons_ = 314.22 ms, *SD* = 34.57 ms; *M*_Instruction_ = 337.66 ms, *SD* = 39.65 ms). If anything, RTs in the compatible condition, *M* = 327.57 ms, *SD* = 40.05 ms, were higher than the incompatible condition, *M* = 326.05 ms, *SD* = 38.33 ms, *t*(53) = 1.58, *p* = 0.06, BF_01_ = 2.10.

To further evaluate the evidence for the absence of a compatibility effect, the compatibility effect (CE) was calculated for all participants regardless of Instructions, *M*_CE_ = −1.515 ms, *SD*_CE_ = 7.05 ms. A directional *T*-Test, *t*(53) = −1.58, *p* = 0.94, BF_0__+_ = 16.31, provided strong evidence for the null hypothesis (i.e., there is no difference between compatible and incompatible condition, namely, CE = 0) against the one-sided alternative hypothesis (i.e., the reaction time in the incompatible condition is longer than the compatible condition, namely, CE > 0).

##### Manipulation check of R-O mappings

Most participants (nearly 91% correct, 49 out of 54) were able to explicitly report the correct mapping of actions and outcomes they were exposed to in the acquisition phase. Participants could still recall previous learned R-O mapping rule. In the No-Instruction condition, 4 out of 25 failed, and only one missed in the Instructions condition (*n* = 29). Collectively, this suggests that participants indeed acquired R-O knowledge spontaneously, although this did not translate into automatic response priming in the test phase.

##### Manipulation check of instructions

In order to assess whether there were differences in how people represented the relation between responses and outcomes in the acquisition phase, the average of each three questions measuring association, prediction, causality, and agency was calculated. The 2 (Instructions condition: No-Instructions vs. Instructions) ^∗^ 4 Representation levels ANOVA only found a main effect of Representation level, *F*(3,156) = 3.69, *p*_[GG]_ = 0.03, $\eta {}_{\text{p}}^{2}$ = 0.07, which showed that collapsed over Instructions conditions, there were significant differences between the questions of the four levels. among these four levels (see Table 1 for more details).

##### Discussion

The results in the present experiment did not reveal a compatibility effect in any of the two groups, suggesting that the presented outcomes did not trigger associated actions. The paradigm used, though, was designed as the strongest test for automatic action selection, with compatibility being manipulated at the trial level. In such a paradigm, compatibility effects have to arise at the trial level itself, if two stimuli evoke either the same or two conflicting responses. As far as we known, only two articles reported compatibility effects on trial level when using the classical two-phases paradigm (Kühn et al., 2009; Sato and Itakura, 2013). However, we were not able to replicate these effects regardless of whether we provided participants with instructions to pay attention to R-O mappings in the acquisition phase or not.

### Experiment 3b: Replication Trial-Based Interference Ideomotor Test

To make sure that the null findings in the rigorous test of Experiment 3a were not a false negative, we conducted a high-powered replication of the core part of Experiment 3b. Because of practical constraints we could not include the manipulation checks, but we assume based on the previous three experiments that most participants were aware of the correct mapping and that the instructions had no effect on the way participants represented the R-O relations.

#### Method

##### Participants and design

Two hundred and two participants (*N* = 202) took part in the experiment in exchange for a small monetary payment or extra course credits. Participants with attention-related disorders or those who were on related medication were excluded beforehand. The experimental design consisted of one between-subjects factor: Instructions (No-Instructions vs. Instructions), and one within-subjects factor: Compatibility (Compatible vs. Incompatible). After signing the informed consent, participants were randomly assigned to either the Instructions condition or the No-Instructions condition.

Data of ten participants were lost because of a technical issue, and six participants were excluded due to the unbalanced proportion of key presses during the learning phase (outside of the range of a left-to-right ratio of 40 to 60%), which was defined before data collection. Data of the remaining 186 participants were analyzed in the test phase (No-Instructions condition: a total of 90 participants, 63 female, age: *M* = 23 years, *SD* = 5; Instructions condition: a total of 96 participants, 70 female, age: *M* = 23 years, *SD* = 4).

##### Stimuli and procedure

We used the same stimuli and procedure as mentioned in Experiment 3a, except that the procedure only had an acquisition phase and a test phase. In this experiment the experimenter was also blind to the real research goals, and waited outside the testing room.

##### Acquisition phase

The acquisition phase was as identical to the one used in Experiment 3a (see Figure 1A).

##### Test phase

The acquisition phase was as identical to the one used in Experiment 3a (see Figure 6).

##### Data analysis plan

Analyses were the same as Experiment 3a, RTs and error rates in the test phase were analyzed as a function of Instructions and Compatibility conditions.

#### Results

##### Acquisition phase

First, we exclude all acquisition trials with omissions (No-Instructions:0.10%, Instructions: 0.08%) and anticipations (No-Instructions:0.15%, Instructions: 0.12%). The remaining mean error rates for the No-Instructions condition was 5.29%, whereas for the Instructions condition it was 5.26%. After that, response proportion (left vs. right keypress) were calculated for each group. The mean left/right response proportions were equal in each condition (No-Instructions condition: 49.8% vs. 50.2%; Instructions condition: 49.5% vs. 50.5%).

The mean RTs of the participants did not differ between the No-Instructions, *M* = 360.76 ms, *SD* = 52.70 ms, and Instructions condition, *M* = 356.10 ms, *SD* = 44.84 ms, *F*(1, 184) = 0.43, *p* = 0.51. The mean RTs of left responses *M* = 356.95 ms, *SD* = 49.96 ms, were significantly faster than the mean RTs of right responses *M* = 359.76 ms, *SD* = 47.69 ms, *F*(1, 184) = 4.91, *p* = 0.03, $\eta {}_{\text{p}}^{2}$ = 0.03. This effect was not qualified by an interaction with the between-subjects factor Instructions, *F*(1, 184) = 0.05, *p* = 0.83.

##### Test phase

Participants who failed to meet the response criteria in the acquisition phase were excluded (six participants). Furthermore, trials with response anticipations (No-Instructions condition: 0.0%, Instructions condition: 0.02%) and omissions (No-Instructions condition: 0.08%, Instructions: 0.11%) were excluded from data analysis.

###### Error rates

Error rates were analyzed based on all valid trials. Similar to the results in Experiment 2b, participants were relatively accurate (*M*_no–instruction_compatible_ = 0.0578, *SD* = 0.073; *M*_no–instruction_incompatible_ = 0.059, *SD* = 0.073; *M*_instruction_compatible_ = 0.0512, *SD* = 0.062; *M*_instruction_incompatible_ = 0.0588, *SD* = 0.090). We employed the same 2-way mixed ANOVA with Instructions as between-subjects factor (No-Instruction vs. Instruction) and Compatibility as within-subjects factor (Compatible vs. Incompatible). Again, the results were not significant: Instructions: *F*(1,184) = 0.11, *p* = 0.74; Compatibility: *F*(1,184) = 2.20, *p* = 0.14; Interaction: *F*(1,184) = 1, *p* = 0.32.

Following the analyses in Experiment 3a, we also conduct the same Bayesian one sample *T*-test for the compatibility effect on error rates by collapsing over the Instructions factor (*M*_error rates_CE_ = 0.0046, *SD*_error rates_CE_ = 0.042). The corresponding BF indicates more support to the null hypothesis (i.e., there is no difference between compatible and incompatible condition, namely, CE = 0), *t*(185) = 1.516, *p* = 0.07, BF_0__+_ = 2.13).

###### Reaction times

The mean RTs on each condition (*M*_no–instruction_compatible_ = 341.29 ms, *SD* = 56.13; *M*_no–instruction_incompatible_ = 341.34 ms, *SD* = 54.61; *M*_instruction_compatible_ = 339.36 ms, *SD* = 52.15; *M*_instruction_incompatible_ = 338.97 ms, *SD* = 53.62) were also subjected to the same 2 (Instructions: No-Instructions vs. Instructions) ^∗^ 2 (Compatibility: Compatible vs. Incompatible) ANOVA, with Instruction as between-subjects and Compatibility as within-subjects factors. No effects approached significance [Instructions: *F*(1,184) = 0.07, *p* = 0.79; Compatibility: *F*(1,184) = 0.04, *p* = 0.84; Interaction: *F*(1,184) = 0.07, *p* = 0.79].

To further evaluate the evidence for the absence of a compatible effect, the CE was calculated for all participants regardless of Instructions (*M*_CE_ = −0.179 ms, *SD*_CE_ = 11.49), and the Bayesian one sample *T*-test still give strong evidence for the null hypothesis (H_0_: CE = 0), *t*(185) = −0.213, *p* = 0.584, BF_0__+_ = 14.34).

#### Discussion

The results in present experiment provide a powerful replication of the effects obtained in Experiment 3a, namely strong evidence for the absence of a compatibility effect and no effects of the Instruction manipulation.

## General Discussion

Habits are often understood as actions that are automatically triggered by stimuli or situations through S-R associations resulting from repeated and consisted coactivation. In the present paper, we explored whether repeated and consistent coactivation of actions and effects can result in similar structures (R-O associations) by which mere perception of stimuli can then elicit the associated response (i.e., ideomotor action). Specifically, we investigated whether learning of R-O associations can occur spontaneously and whether as a result, these stimuli can automatically trigger associated responses. Accordingly, in four experiments, we tested automaticity in ideomotor learning in the standard two-phases paradigm that required participants to perform actions (pressing keys) that lead to specific outcomes (tones). In each experiment, we manipulated instructions in a free-choice learning phase, either making no mention in any way of the tones that followed actions, or induced a processing goal that explicitly emphasizing the relation between responses and the subsequent stimulus. In Experiment 1, evidence for ideomotor action was observed in a free-choice test phase, regardless of instructions. Experiment 2, 3a, and 3b, however, which employed forced-choice tasks to test for automaticity, provided little evidence for ideomotor effects. Together, these results don’t support the strong version of ideomotor theory. That is, they suggest that ideomotor learning can occur spontaneously, but that there are limits to the automatic effect on behavior.

### Mixed Evidence for Automatic Ideomotor Effects

The findings of Experiment 1 demonstrate that ideomotor learning can take place in the absence of explicit instructions that emphasize the relation between actions and outcomes. Although this finding matches with the literature on implicit learning (e.g., Cleeremans et al., 1998) and may indicate that associations have been formed as a result of coactivation of response and resulting stimulus representations, this does not necessarily mean that learning occurred outside of awareness (Melnikoff and Bargh, 2018). Indeed, given the fact that the large majority of participants could indicate which outcome was produced by which action in the acquisition phase, and the relatively high scores on these R-O mapping checks in the no-instruction and instruction conditions, it seems to be the case that although learning was spontaneous and may have resulted in associations, the acquired knowledge was clearly propositional in nature (Mitchell et al., 2009). This effect on the R-O mapping checks was consistent across all experiments, although reports were understandably less accurate when the mapping was changed during the test phase in Experiment 2. In sum, while learning occurred spontaneously, it seems that participants had explicit knowledge about which action caused which outcome in the learning phase.

The results of the different test phases across our experiments at first seem liked a mixed bag. While Experiment 1 produced a healthy ideomotor effect consistent in size with the ideomotor literature (c.f., Elsner and Hommel, 2001), Experiments 2, 3 did not provide such evidence. An exception is the effect in Experiment 2 on error rates in the first block of trials of the test phase. Below, we entertain two possible explanations to reconcile these findings.

First, one could argue that on top of the reportable causal knowledge about the action outcome mappings, people did indeed form bi-directional associations, capable of producing ideomotor effects. This explanation is consistent with the findings of Experiment 1. In accordance with the strong version of ideomotor theory (Shin et al., 2010), merely hearing the tones during the free-choice task could have automatically triggered the associated responses, leading to more mapping-consistent responses. As the learning phases and explicit reports were quite similar in Experiments 2 and 3, one would have to assume, though, that the tones at least had the potential to trigger similar responses in the corresponding test phases. Maybe the null effects there could be explained by a lack of power. This seems unlikely, though, as the number of trials is comparable with other reports in the ideomotor literature and the failure to find an effect in the high-powered replication of Experiment 3 seems more in line with the absence of an effect. It could be the case that the test tasks in Experiments 2, as well as Experiment 3a and its replication, were somehow flawed and not able to pick up the ideomotor effect. This seems unlikely as well. The tasks were closely modeled after Elsner and Hommel (2001) and should theoretically have produced the ideomotor effect, at least according to the strong version of the theory.

A theoretical explanation for the null effects in Experiment 2 and 3, though, is that people were able to suppress or inhibit ideomotor responses in the test phase. It has recently been argued that automatic responding may emerge in some tasks, but be overruled in others in which people have the goal to inhibit such responses (Melnikoff and Bargh, 2018). Although the task instructions in the test phases of Experiments 2 and 3 did not explicitly ask people to ignore the tones, it may be the case that people tried to ignore them, or at least suppress responses in order to meet the task goal. That is, responding according to the dictated response rule (Experiment 2), or responding to the visual target (Experiment 3 and its replication). It could indeed be possible that people were able to inhibit ideomotor responses in the task and exactly cancel out the effect, without revealing an opposite inhibition effect, or were fully able to shut out the auditory stimuli in the compatibility tasks, but not the free choice task. However, we believe another explanation is more plausible.

This second explanation follows the opposite line of argument: that bi-directional associations were not formed, at least not strong enough for the tones to trigger responses in an automatic fashion. This would then require and explanation for the findings in Experiment 1. In this experiment, participants engaged in a free-choice task, which – by definition – allows for deliberate control of behavior. It may have been the case that explicit knowledge about the action-outcome relations drove the behavioral effects (Seabrooke et al., 2016). Loersch and Payne (2011) have noted that such biases can occur if primes affect the explicit knowledge that is retrieved and used as input for the decision-making process. Although this does not necessarily imply that participants were aware of this bias, it would entail an indirect priming effect that operates through biasing conscious decisions rather than by stimuli automatically triggering responses. Although this may suggest that people use knowledge of R-O mappings to freely select their actions, this would not be ideomotor action according to the strong version of the theory. Interestingly, though, such a process fits well with action control models that consider the preparation of human behavior to be rooted in sensorimotor processes that operate under radar of conscious awareness, while the ultimate execution of actions is under the control of a decision making process that selects actions associated with an act of conscious will (Gold and Shadlen, 2007; Brass and Haggard, 2008; Aarts, 2012; Zedelius et al., 2014).

Another explanation for the findings of Experiment 1 is that participants may have used the tones to fulfill the criteria of responding randomly and equally often with the two keys, or have chosen to respond with the keys suggested by the tones simply because it is easier. Random selection of responses is extremely hard and the tones may have provided an easy way out. Note that this explanation still assumes that people use the R-O knowledge that was spontaneously obtained in the acquisition phase. As a considerable number of participants responded consistent with the mapping of the acquisition phase on nearly 100% of the trials (and two individuals in close to 0% of the time, reflecting the use of a reversed mapping; see Figure 2), this seems a plausible explanation. Although papers in the ideomotor literature typically don’t provide information about the distribution of scores, the means and standard deviations in the present study are remarkably similar to earlier studies (e.g., Elsner and Hommel, 2001) suggesting that these studies may be open to the same explanation.

In Experiment 2, we found no within-participants compatibility effects, but did obtain a difference in error rates in the first block of the experiment. While this effect is consistent with the classic forced-choice effect (e.g., Elsner and Hommel, 2001, Experiments 1a, 1b), these effects could also be interpreted as a task-switching effect (Monsell, 2003). That is, in the light of the explicit knowledge about the R-O mapping in the acquisition phase, the instruction to use the opposite mapping to respond to the outcome stimuli in the test phase could have caused the increase in errors. Hence, the obtained compatibility effect may say more about the challenges of remembering and responding according to reversed task rules, than ideomotor effects. The complexity of obtaining ideomotor effects under forced-choice conditions (Herwig et al., 2007; Pfister et al., 2011), and the relative absence of ideomotor effects in the forced-choice task in the present study indicates that further inquiry is needed to specify when and how ideomotor learning effects emerge in the test paradigms employed so far.

### Implications for Habits

Although ideomotor learning can create R-O associations, only weak evidence for the ideomotor effect was obtained. So based on the current data, it seems that S-R associations underlying habits function in a different way than the R-O learning that drove the ideomotor effect in our free-choice test phase in Experiment 1. This does not necessarily mean that ideomotor action should be discarded as a mechanism by which outcome stimuli can trigger responses, in a similar way as stimuli trigger habitual responses. As the ideomotor effect has been demonstrated across a large literature (although often with less strict tests than in the current experiments), it could be the case that the ideomotor effect holds, but that the learning phase in our experiments was too short for R-O associations to develop through co-activation, and that habit-like structures take longer to develop. Moreover, research on rewards in ideomotor learning has demonstrated that rewarding stimuli that follow responses produce much stronger ideomotor effects in free-choice or instructed compatibility tasks (Muhle-Karbe and Krebs, 2012; Eder et al., in press). It may be the case that ideomotor learning is therefore more likely to occur in daily life, where stimuli following actions are rarely neutral. Interestingly, with this notion, the ideomotor effect becomes similar to the Pavlovian Instrumental Transfer (PIT) effect, which holds that stimuli associated with rewards are found to facilitate instrumental responses that have been followed by those rewards during learning (Watson and de Wit, 2018). Such a mechanism may reflect habitual responses that are still mediated by outcome representations at some level.

Further research is needed, though, to determine how rewards boost responses in the ideomotor and PIT paradigm. As ideomotor studies on this topic (Muhle-Karbe and Krebs, 2012; Eder et al., in press) used a block-based compatibility paradigm, the enhanced effects could still be the results of explicit knowledge, as a result of propositional learning, interfering with conflicting task instructions. Relatedly, recent investigations into the nature of the PIT effect have demonstrated that the PIT effect itself is also dependent on propositional learning (Trick et al., 2011; Seabrooke et al., 2016, 2017). As here it is also unclear whether rewards influence learning, response execution, or both, it is hard to predict whether the same results would emerge in a trial-based compatibility task, to provide strong evidence for the automaticity of ideomotor action.

### Conclusion

Together, while the current findings do provide evidence for spontaneous ideomotor learning, it is less evident how resulting response-stimulus representations subsequently guide behavior. Rather than automatically facilitating responses, it may be the case that R-O knowledge affects behavior in a less automatic way. While primed outcomes may activate knowledge of associated actions (Bargh et al., 2001; Custers and Aarts, 2005, 2010), they may influence behavior indirectly by biasing conscious choice (see e.g., Custers et al., 2012). As such, responses following outcome primes may be more the result of biased choice than direct response priming. Given the parallels between ideomotor thinking and the study of habitual behavior, the current work suggests that research on habitual behaviors could benefit from more careful experimentation and theorizing (Marien et al., 2018, 2019) to help understand in which ways cues in the environment could elicit habitual behavior.

## Data Availability Statement

The raw data supporting the conclusions of this article will be made available by the authors, without undue reservation, to any qualified researcher.

## Ethics Statement

This study was carried out in accordance with the recommendations of the principles of the Declaration of Helsinki and the Dutch Code of Conduct for Scientific Practices as determined by the VSNU Association of Universities in Netherlands, Faculty Ethics Review Board (FERB) of the Faculty of Social and Behavioral at Utrecht University with written informed consent from all subjects. All subjects gave written informed consent in accordance with the Declaration of Helsinki. The protocol was approved by the Faculty Ethics Review Board (FERB) of the Faculty of Social and Behavioral at Utrecht University.

## Author Contributions

DS, RC, and HA designed the study. DS collected and analyzed the data. DS, RC, HM, and HA drafted the manuscript. RC and HM provided the critical revisions. All authors approved the final version of the manuscript for submission.

## Conflict of Interest

The authors declare that the research was conducted in the absence of any commercial or financial relationships that could be construed as a potential conflict of interest.

## Footnotes

1 Cohen’s dz is the standardized mean difference effect size, for a detailed calculation see Lakens (2013).
