# Supplementary material for: Ideomotor Action: Evidence for Automaticity in Learning, but Not Execution
Source: Front Psychol. 2020 Feb 14;11:185. doi: 10.3389/fpsyg.2020.00185 (PMC7033682; doi:10.3389/fpsyg.2020.00185)
Supplement: Supplementary file 1 [file Table_1.DOCX]

Supplementary Materials

# Supplementary Figures and Tables

## Supplementary Figures for representation checks in each experiment

##
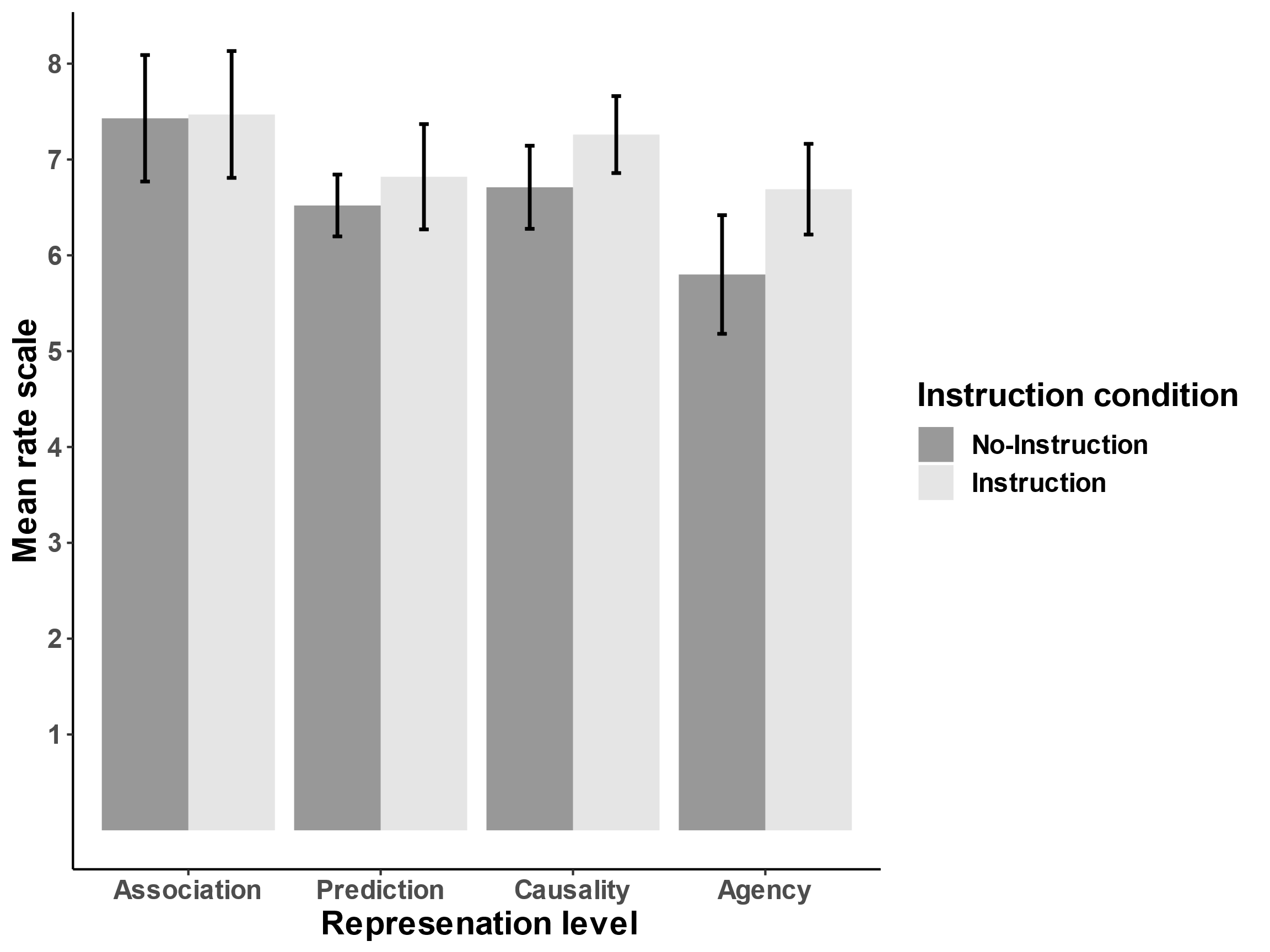


**Supplementary Figure 1.1** Results of the manipulation check of Instructions for Experiment1: Free-choice. The bars represent the means of the four representation levels as a function of Instructions condition. Note: Error bars represent 95% confidence intervals of the means.


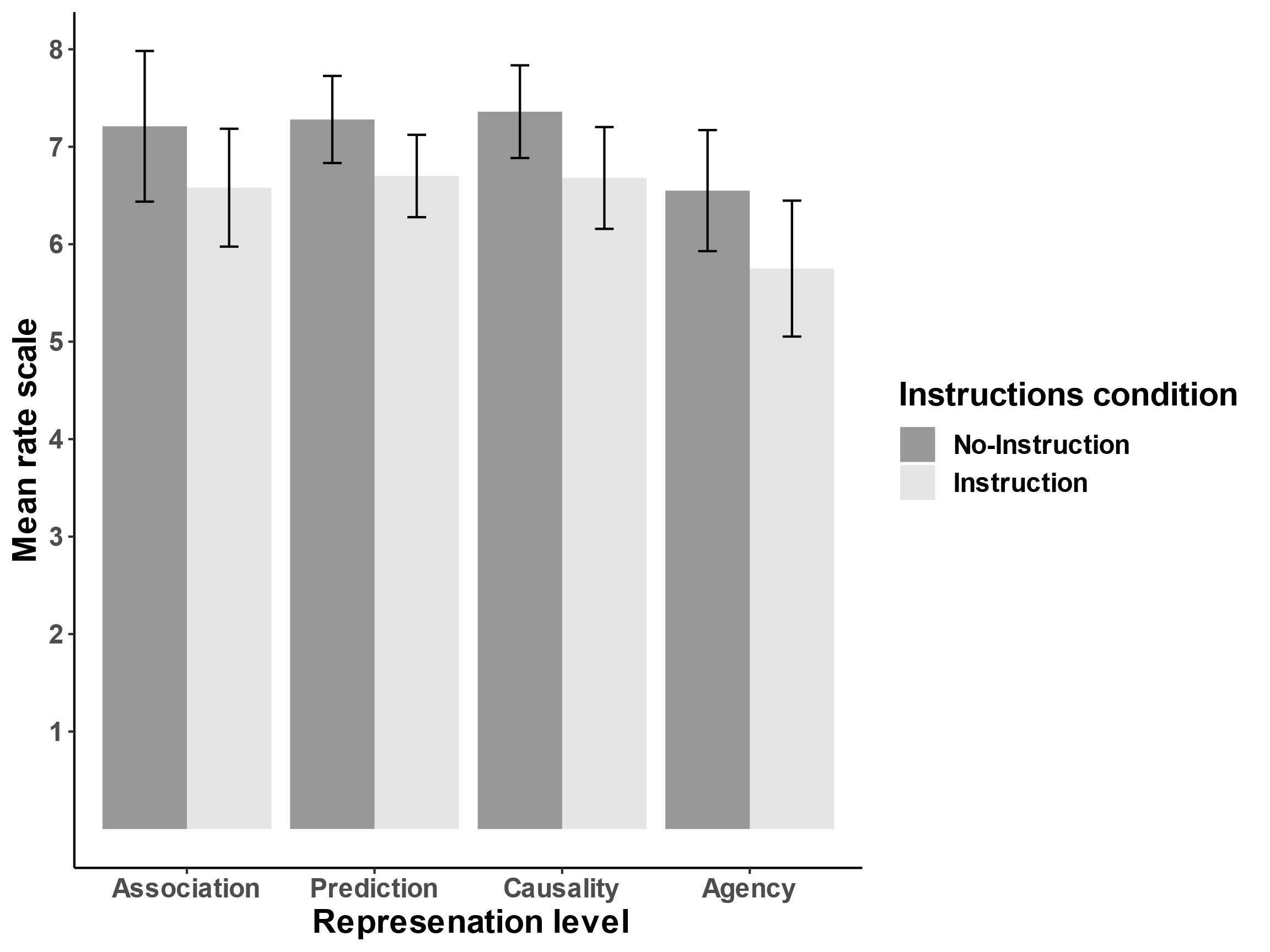


**Supplementary Figure 1.2.** Results of the manipulation check of Instructions for Experiment 2:Block-based Interference ideomotor test. The bars represent the means of the four representation levels as a function of Instructions condition. Note: Error bars represent 95% confidence intervals of the means.


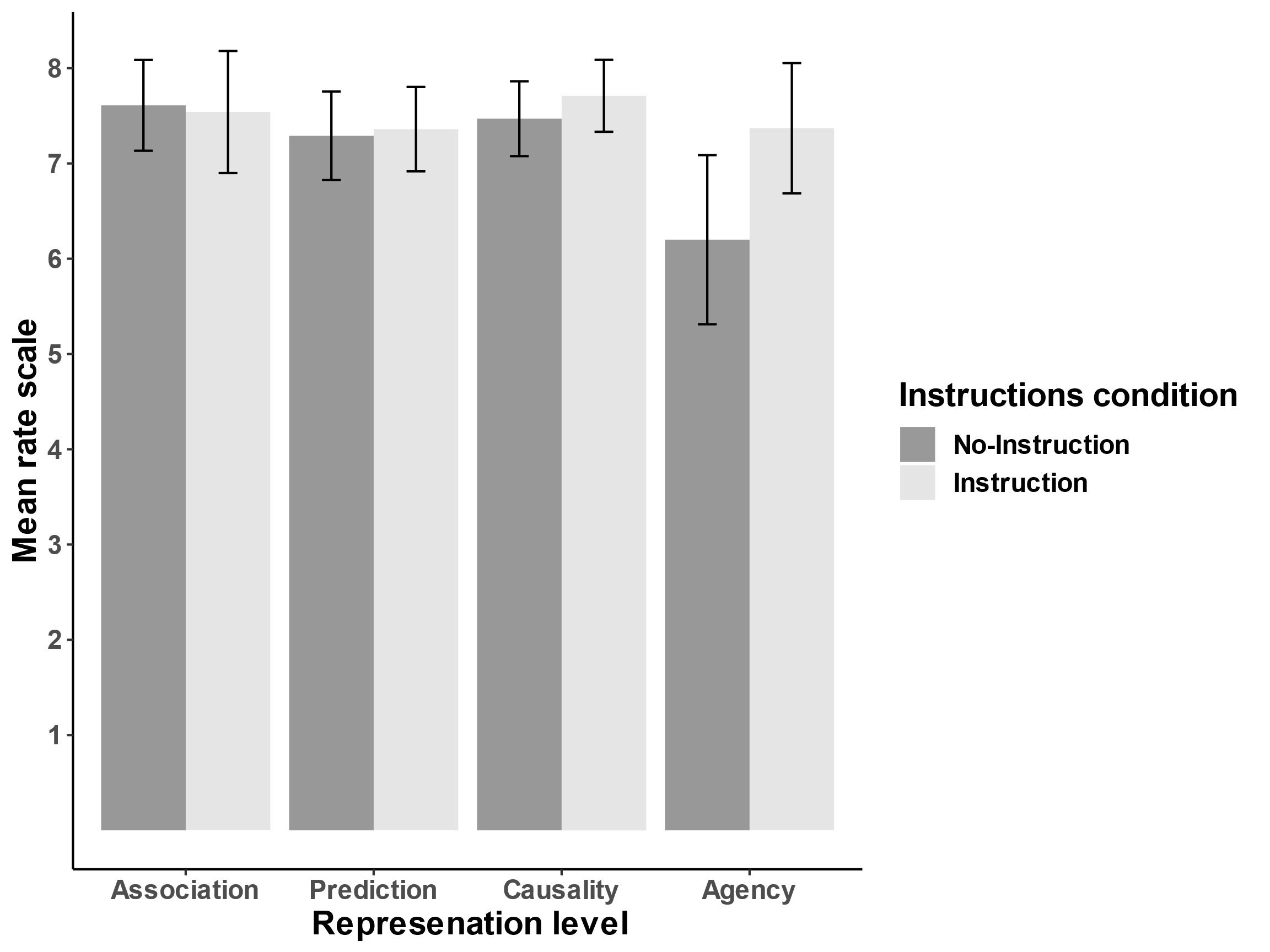


**Supplementary Figure 1.3.** Results of the manipulation check of Instructions for Experiment 3a: trial-based. The bars represent the means of the four representation levels as a function of Instructions condition. Note: Error bars represent 95% confidence intervals of the means.

## Supplementary Figures of correlation matrix for each Experiment


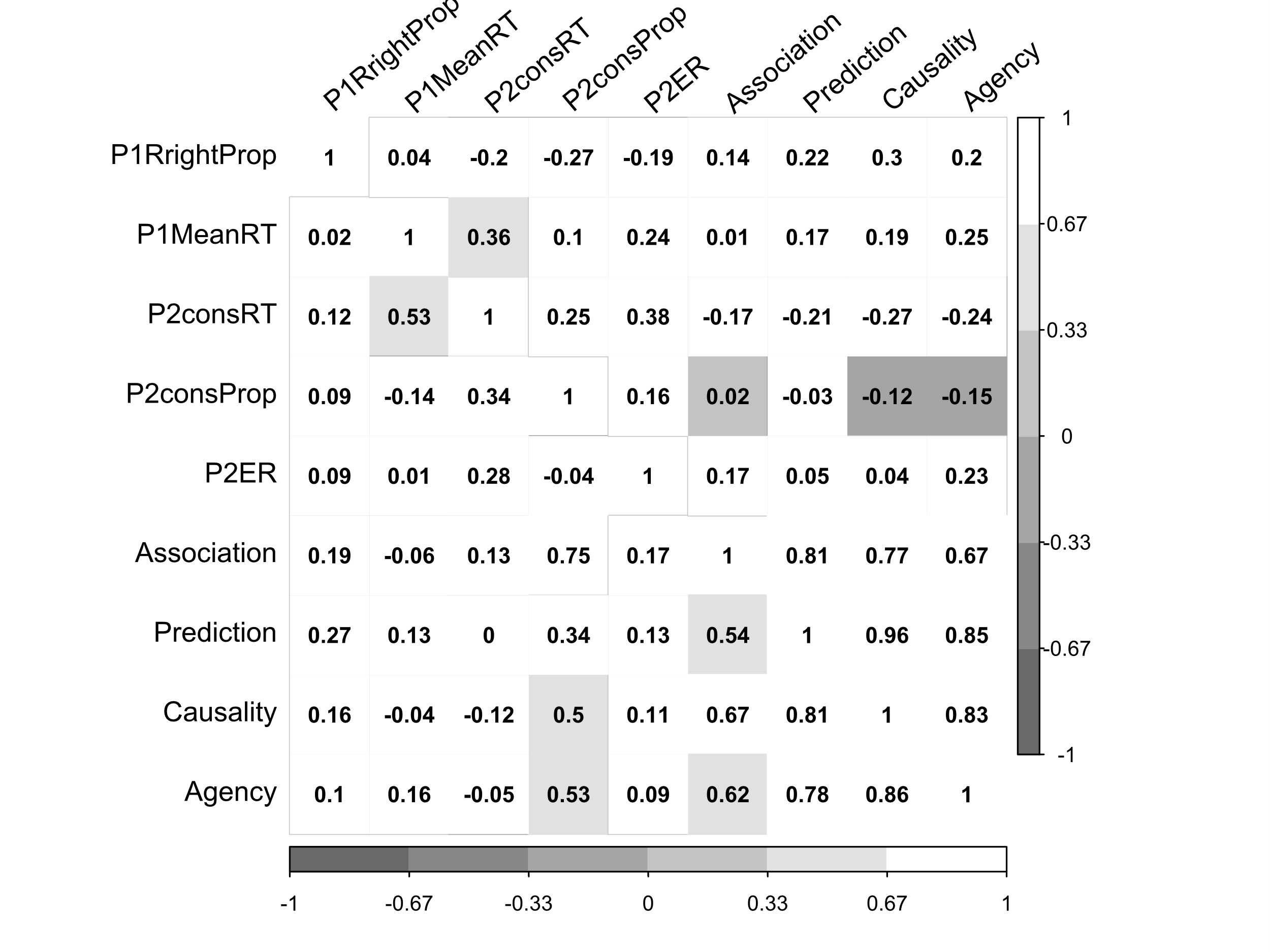


**Supplementary Figure 2.1** The correlation between RTs in the acquisition phase and (a) performance of test phase, including consistent proportion, consistent RTs, and (b) representation levels in the manipulation check of Instructions, that is , Association, Prediction, Causality and Agency in Experiment 1: free-choice. (1) Separating from the right diagonal of the square, the lower part is the correlation for Instruction group, and the upper part for No-Instruction group. Coefficient correlations are the values inside the square. We set the threshold of significance to 0.05, and only significant correlation are highlighted in gradient colors. (2).labels are the abbreviation of the related variables in the acquisition phase (P1) and the testing phase (P2): P1RightProp – the proportion of right hand in the acquisition phase; P1MeanRT – the mean reaction times in the acquisition phase; P2consRT – reaction times of consistent / compatible condition in the testing phase; P2consProp – proportion of consistent / compatible condition in the testing phase; P2ER – error rates in the testing phase.


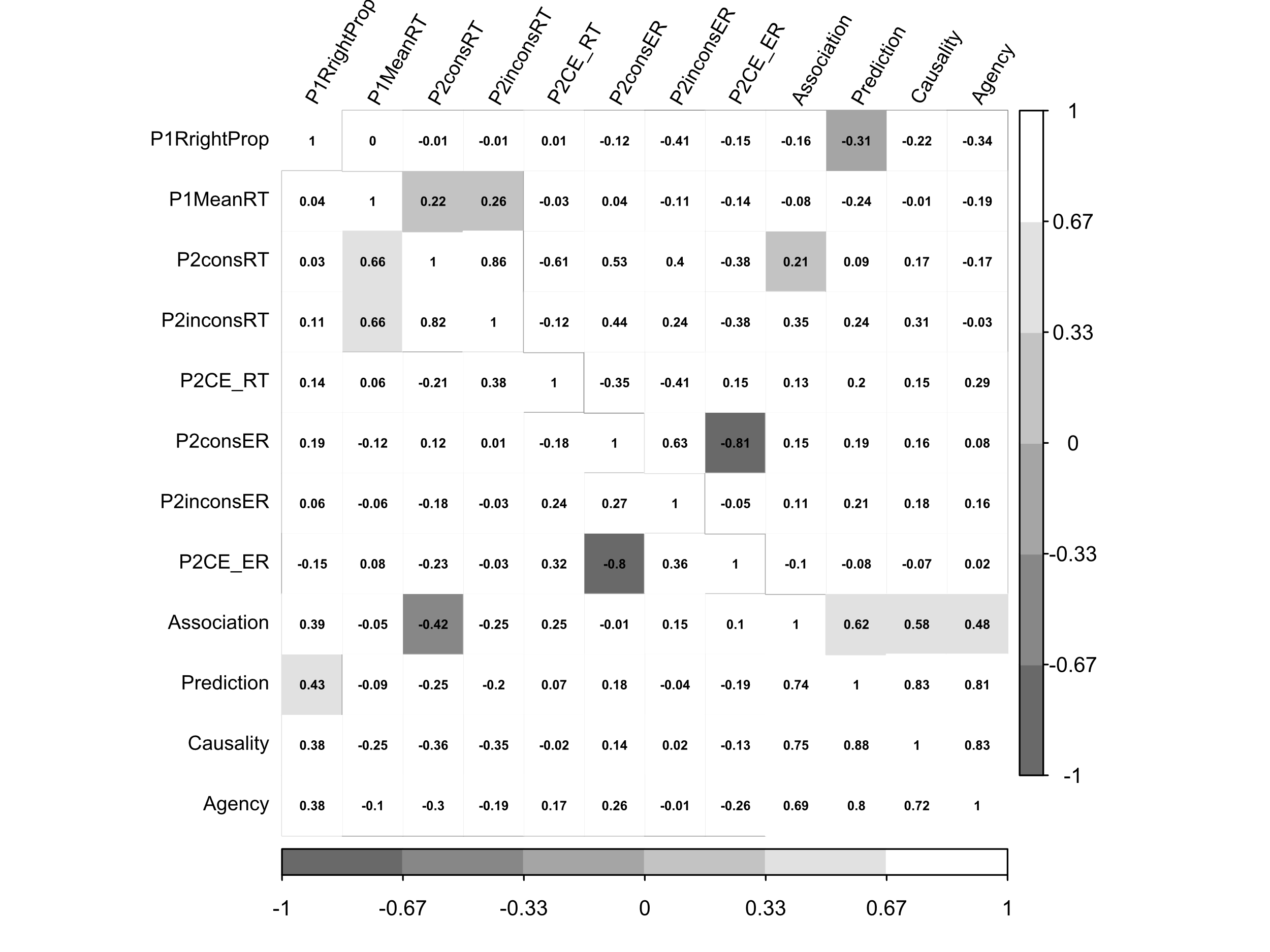


**Supplementary Figure 2.2.** The correlation between RTs in the acquisition phase and (a) performance of test phase, including consistent proportion, consistent RTs, and (b) representation levels in the manipulation check of Instructions, that is , Association, Prediction, Causality and Agency in Experiment 2: block-based. (1) Separating from the right diagonal of the square, the lower part is the correlation for Instruction group, and the upper part for No-Instruction group. Coefficient correlations are the values inside the square. We set the threshold of significance to 0.05, and only significant correlation are highlighted in gradient colors. (2).labels are the abbreviation of the related variables in the acquisition phase (P1) and the testing phase (P2), and are the same as used in Supplementary Figure 4, except that CE was the performance (RT or ER) subtracted consistent condition from inconsistent condition.


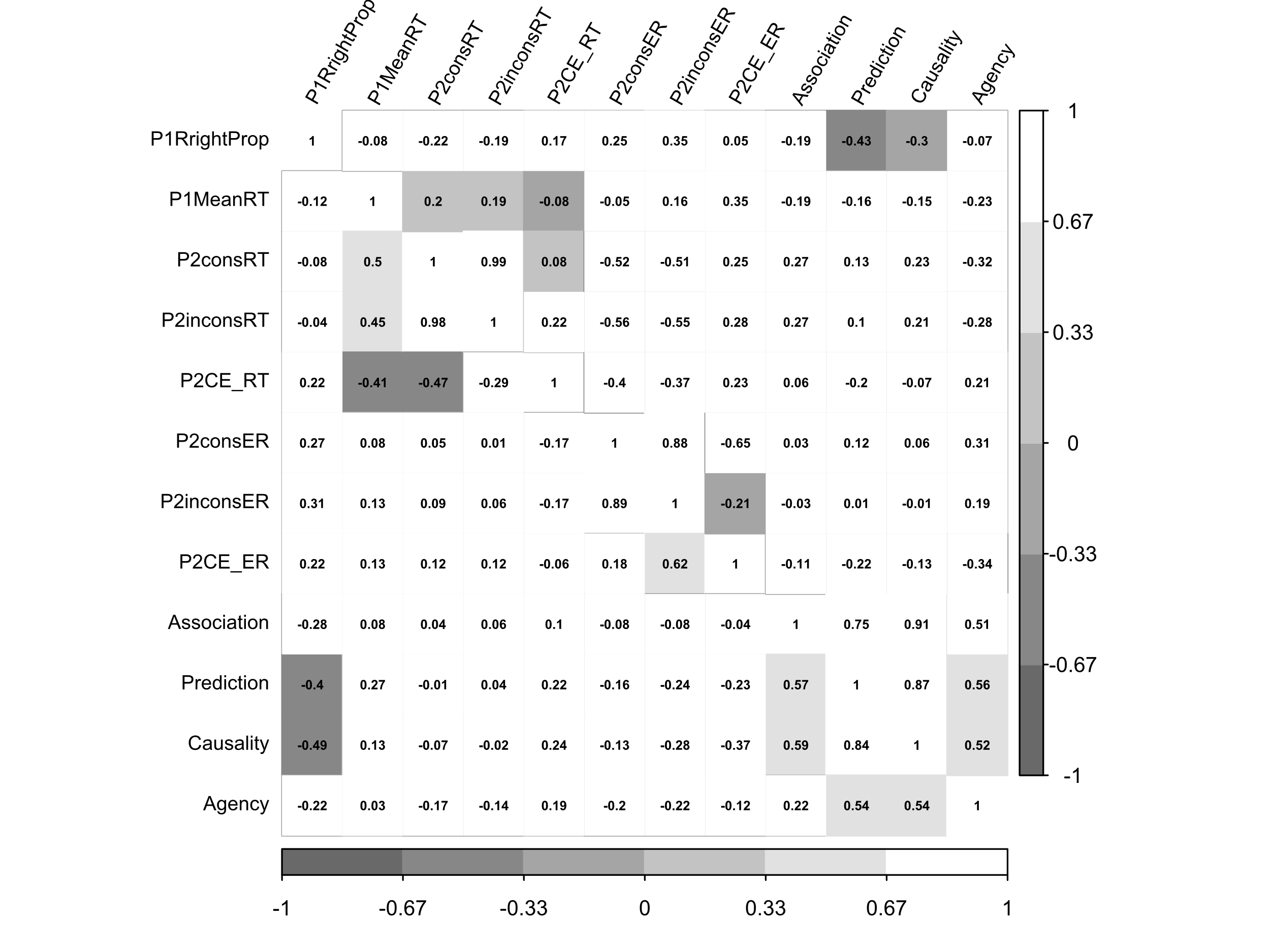


**Supplementary Figure 2.3.** The correlation between RTs in the acquisition phase and (a) performance of test phase, including consistent proportion, consistent RTs, and (b) representation levels in the manipulation check of Instructions, that is , Association, Prediction, Causality and Agency in Experiment 3a: trial-based. (1) Separating from the right diagonal of the square, the lower part is the correlation for Instruction group, and the upper part for No-Instruction group. Coefficient correlations are the values inside the square. We set the threshold of significance to 0.05, and only significant correlation are highlighted in gradient colors. (2).labels are the abbreviation of the related variables in the acquisition phase (P1) and the testing phase (P2), and are the same as used in Supplementary Figure 3.1.

## Supplementary Tables of descriptive tables for each Experiment

Supplementary Table 1.1 Descriptive Info of Experiment 1(Mean and SD)

|  | Acquisition phase (P1) | | | Testing phase (P2) | | |
| --- | --- | --- | --- | --- | --- | --- |
|  | Right Prop. | Left RT. | Right RT. | cons. Prop | cons. RT | Error rate |
| No-Instruction | 50.13  (2.53) | 365.88  (56.89) | 360.01  (64.55) | 61.49  (22.61) | 502.42  (95.89) | 1.77  (2.30) |
| Instruction | 50.38  (1.98) | 363.36  (40.04) | 361.46  (38.77) | 69.98  (25.42) | 514.99  (70.24) | 2.20  (5.23) |

Note: For all Experiments, the acquisition phase (P1) used the same paradigm, that is, the free choice task, which means that the main dependent variable for the acquisition phase is proportion (%) of between the Left hand and Right hand with Instruction (No-Instruction vs. Instruction) as a between-subject factor. In Experiment 1, we used a free-choice Ideomotor task for the testing phase (P2), and then compared the percentage of responses that were consistent (cons.Prop) with the previously acquired Response-Outcome mapping and error rates (ER)(%). For exploratory analyses, we also compared the RTs between consistent and inconsistent condition.

Supplementary Table 1.2 Descriptive Info of Experiment 2 (Mean and SD)

|  | Acquisition phase (P1) | | | Testing phase (P2) | | | | | |
| --- | --- | --- | --- | --- | --- | --- | --- | --- | --- |
|  | Right Prop. | Left RT. | Right RT. | comp. RT | incom. RT | CE.  RT | comp ER | incom. ER | CE.  ER |
| No-Instruction | 49.78  (1.79) | 375.05  (37.08) | 373.72  (31.37) | 486.21  (131.69) | 481.96  (126.94) | -6.96  (43.94) | 3.49  (6.27) | 3.16  (3.67) | -0.33  (0.05) |
| Instruction | 50.36  (1.34) | 375.13  (39.43) | 378.01  (36.77) | 457.52  (117.77) | 459.58  (122.40) | 1.85  (41.09) | 2.94  (3.32) | 3.27  (2.12) | 0.33  (0.03) |

Note: In Experiment 2, we used a block-based Ideomotor task for the testing phase. The order between compatible and incompatible block did not take into consideration. We compared the RTs (ms) and ERs (%) with Instruction (No-Instruction vs. Instruction) as a between-subject factor and compatibility (compatible vs. incompatible) as a within-subject factor [CE = Incompatible – compatible].

Supplementary Table 1.3 Descriptive Info of Experiment 3a (Mean and SD)

|  | Acquisition phase (P1) | | | Testing phase (P2) | | | | | |
| --- | --- | --- | --- | --- | --- | --- | --- | --- | --- |
|  | Right Prop. | Left RT. | Right RT. | comp.  RT | incom.  RT | CE.  RT | comp.  ER | incom.  ER | CE.  ER |
| No-Instruction | 50.40  (1.18) | 348.35  (53.52) | 340.87  (49.87) | 314.48  (34.55) | 313.95  (35.30) | -0.53  (5.09) | 5.00  (7.02) | 5.33  (5.44) | 0.33  (2.51) |
| Instruction | 50.18  (1.87) | 360.12  (43.79) | 356.01  (43.85) | 338.85  (41.58) | 336.48  (38.33) | -2.37  (8.38) | 3.54  (4.29) | 4.26  (5.35) | 0.72  (3.42) |

Note: In Experiment 3a, the information reported at the acquisition phase follow those of Experiment 1, except that we used a trial -based Ideomotor task for the testing phase. We compared the RTs (ms) and ERs (%) with Instruction (No-Instruction vs. Instruction) as a between-subject factor and compatibility (compatible vs. incompatible) as a within-subject factor. [CE = Incompatible – compatible]

# Appendix

## Manipulation check of R-O mappings

Dear participant,

Congrats! You have almost reached the end of the experiment. We will ask you to answer a few more questions only about the first part.

Please Remember:

The following questions are based on first part(P1).

Which tone did the left key press produce?

① the left key press produced the High tone

② the left key press produced the Low tone

③ the left key press produced both tones

④ the left key press was irrelevant to the tones

Which tone did the right key press produce?

① the right key press produced the High tone

② the right key press produced the Low tone

③ the right key press produced both tones

④ the right key press was irrelevant to the tones

## Manipulation check of Instructions

(1) Do you think particular tones were associated with particular key presses?

Not at all 1 2 3 4 5 6 7 8 9 very much

(2) Do you think particular tones went together with particular key presses?

Not at all 1 2 3 4 5 6 7 8 9 very much

(3) Do you think particular tones occurred with particular key presses?

Not at all 1 2 3 4 5 6 7 8 9 very much

(4) Could you predict the particular tones based on your key presses?

Not at all 1 2 3 4 5 6 7 8 9 very much

(5) Could you anticipate the particular tones based on your key presses?

Not at all 1 2 3 4 5 6 7 8 9 very much

(6) Did particular tones follow particular key presses?

Not at all 1 2 3 4 5 6 7 8 9 very much

(7) Do you think particular key presses caused particular tones?

Not at all 1 2 3 4 5 6 7 8 9 very much

(8) Do you think particular key presses produced particular tones?

Not at all 1 2 3 4 5 6 7 8 9 very much

(9) Do you think particular key presses generated particular tones?

Not at all 1 2 3 4 5 6 7 8 9 very much

(10) Did you feel you could influence which tone would occur?

Not at all 1 2 3 4 5 6 7 8 9 very much

(11) Did you feel you could control which tone would occur?

Not at all 1 2 3 4 5 6 7 8 9 very much

(12) Did you feel you could determine which tone would occur?

Not at all 1 2 3 4 5 6 7 8 9 very much
